# Effects of nonequilibrium fluctuations on ultrafast short-range electron transfer dynamics

**DOI:** 10.1038/s41467-020-15535-y

**Published:** 2020-06-04

**Authors:** Yangyi Lu, Mainak Kundu, Dongping Zhong

**Affiliations:** 1 Department of Physics, Department of Chemistry and Biochemistry, Programs of Biophysics, Chemical Physics, and Biochemistry, The Ohio State University, Columbus, OH 43210 USA; 2 Center for Ultrafast Science and Technology, School of Chemistry and Chemical Engineering, School of Physics and Astronomy, Shanghai Jiao Tong University, 200240 Shanghai, China

**Keywords:** Biophysics, Molecular biophysics, Chemistry, Photochemistry, Physical chemistry

## Abstract

A variety of electron transfer (ET) reactions in biological systems occurs at short distances and is ultrafast. Many of them show behaviors that deviate from the predictions of the classic Marcus theory. Here, we show that these ultrafast ET dynamics highly depend on the coupling between environmental fluctuations and ET reactions. We introduce a dynamic factor, *γ* (0 ≤ *γ* ≤ 1), to describe such coupling, with 0 referring to the system without coupling to a “frozen” environment, and 1 referring to the system’s complete coupling with the environment. Significantly, this system’s coupling with the environment modifies the reaction free energy, Δ*G*^*γ*^, and the reorganization energy, *λ*^*γ*^, both of which become smaller. This new model explains the recent ultrafast dynamics in flavodoxin and elucidates the fundamental mechanism of nonequilibrium ET dynamics, which is critical to uncovering the molecular nature of many biological functions.

## Abstract (web summary)

Ultrafast electron-transfer reactions are fundamental to protein functions. Here the authors show that these reaction dynamics are affected by the ruggedness of protein energy landscape, which even modifies the reaction free energy and reorganization energy.

## Introduction

The electron transfer reaction is one of the fundamental steps in biological processes^1,2^. Biological electron transfer (ET) reactions show a great variety of dynamics with respect to a wide span of reaction timescales, from femtoseconds (fs) to milliseconds (ms), and a large variation of donor-acceptor distances^3^. These ET dynamics often display behaviors of non-exponential decays^4–6^.

It is widely known that, when environmental fluctuations are much faster than the ET reaction, the ET dynamics can be described by a single exponential decay,

1 $$Q\left( t \right) = e^{ - t/\tau _{{\mathrm{ET}}}},$$

where *Q*(*t*) is understood as the survival probability of reactants. This class of ET reactions has been successfully elucidated by the classic Marcus theory^7^, with the reaction rate given by

2 $$k_{{\mathrm{ET}}} = 1/\tau _{{\mathrm{ET}}} = A\sqrt {\frac{1}{{4\pi k_BT\lambda }}} e^{ - \frac{{\left( {{\mathrm{\Delta }}G^o + \lambda } \right)^2}}{{4k_BT\lambda }}},$$

in which Δ*G*^*o*^ is the free energy of the ET reaction, and *λ* is the reorganization energy. In the nonadiabatic limit, the prefactor *A* is determined by the Franck–Condon principle^8^,

3 $$A = \frac{{2\pi }}{\hbar }J^2,$$

where *J* is the electronic coupling constant between the two states.

However, in some biological ET reactions, such as charge separations in reaction center proteins, the ET dynamics is observed to have the same timescale as those of local fluctuations. There exist a large class of reaction center proteins, known as flavoproteins, which contain the flavin cofactor, in the form of flavin adenine dinucleotide (FAD) or flavin mononucleotide (FMN)^9^. Flavin molecules have three redox states: oxidized, semiquinone (one-electron reduced), and hydroquinone (two-electron reduced). Hence, the flavin molecules can participate in one-electron and two-electron transfer processes, as well as proton-coupled-electron-transfer (PCET) processes^10^. Because of the chemical versatility of flavin molecules, flavoproteins are ubiquitous in biological systems and participate in enzyme-mediated oxido-reduction reactions that are part of many crucial biological functions^11^. The microscopic mechanisms behind these enzyme activities involve a series of short-range ET reactions, of which the donor and acceptor are within a few Å. These ET reactions happen in the ultrafast timescales along with fluctuations of their local environments^12^. Recent work has suggested that the nonequilibrium environment has a major impact on the ultrafast dynamics of short-range ET reactions, which are not clearly understood within the classical framework^13,14^.

**Fig. 1:**
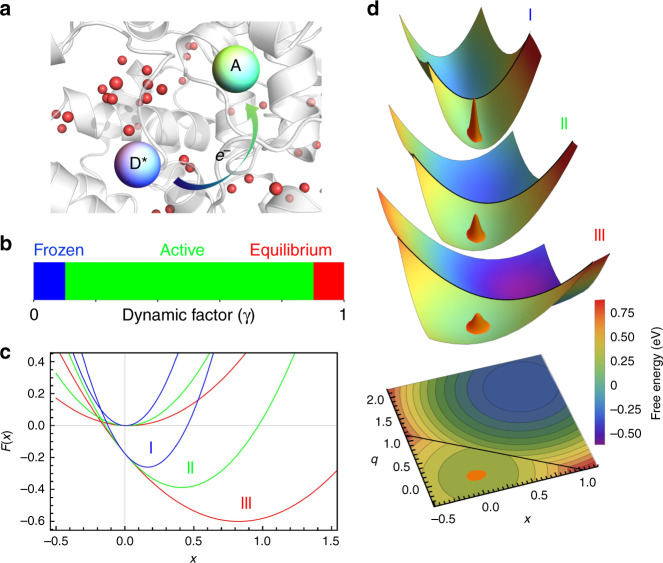
Schematic representations of the ultrafast electron transfer model. **a** Cartoon illustration of a photo-excited electron transfer reaction inside a protein. **b** Three categories of ET reactions characterized by the value of dynamic factor *γ* (Eq. 16). **c** Free energy curves of three categories of ET reactions along the solvent coordinate *x*. Two curves with the same color, corresponding to the colored ET category in **b**, are related to the reactant (left curve) and product (right curve) states of a ET reaction, respectively (Eqs. 10a and 10b). **d** Free energy surfaces of three categories of ET reactions. Golden wavepackets represent the initial distributions, *P*(*x*, *t* = 0) (Eq. 30).

For example, the flavodoxin, being a flavoprotein, is an electron carrier, which is non-covalently bonded to a flavin molecule (FMN)^15^. Upon excitation, the flavin cofactor, originally at the state of semiquinone (FMNH^•^), accepts an electron from an aromatic tryptophan or tyrosine residue (W or Y), and is reduced to the hydroquinone state (Fig. 1a). These reactions happen in the timescale of a few picoseconds and cannot be described by a single-exponential decay. They often show a stretched-exponential behavior

4 $$Q\left( t \right) = e^{ - \left( {t/\tau _{{\mathrm{ET}}}} \right)^\beta },$$

in which *β* is <1.0^16,17^. The solvation dynamics of the flavodoxin, which characterizes local motions of the protein and trapped water, was resolved and described by two components with time constants of a few picoseconds (ps) and tens of picoseconds, respectively^18^.

Interestingly, a different type of ET dynamics arises when the ET reaction is faster than local motions of protein and water^19^. This phenomenon was observed in flavodoxin with the flavin cofactor being prepared at the oxidized state (FMN). In this case, the ET reaction between the residual (W or Y) and the oxidized flavin (FMN) happens within a few hundred femtoseconds upon excitation, which is ahead of the fastest local relaxation measured in the solvation experiment^18^. Surprisingly, these ET dynamics are very well described by single-exponential decays. Similar dynamics has been reported in other flavoproteins^20,21^ and possibly in other classes of proteins^22–24^.

It has been well established that the free energy landscape of a protein in solution is characterized by a large number of local minima, which occupy a hierarchy of energy scales^25–27^. Starting from a random part of the energy landscape, accessing different local minima requires relaxation motions with different timescales, from local orientational motions in fs to protein conformational transitions in ms or even longer^28,29^. If the time window is limited (for example, by the ET reaction time), the complete phase space is not fully sampled. In other words, ergodicity is broken for this type of systems and the traditional approach of thermodynamics is not applicable^30^.

It is reasonable to expect that the “degree of ergodicity breaking” of a ET system is determined by the ET reaction timescale. Depending on the relative timescales of ET reactions and the corresponding environmental relaxations, these reactions are categorized into three classes (Fig. 1b):

1. Frozen: the reaction is faster than any local motion of the environment. The surrounding protein and water do not have time to respond to the charge transfer between the donor and acceptor within the reaction time window. That is, the ET happens in a “frozen” environment^19^.
2. Active: the ET reaction is actively coupled to environmental relaxation modes. This class exhibits a variety of dynamics, which typically displays non-exponential behaviors^6^.
3. Equilibrium: the ET reaction is much slower than local environmental fluctuations such that the populations of reactants and products are always at equilibrium with the environment. This type of ET is finely explained by the Marcus theory^31^.

In this work, in order to understand the novel dynamics observed in ultrafast short-range ET reactions, we propose a generalized reaction-diffusion model, which incorporates the effect of ergodicity breaking in the model developed by Sumi and Marcus^32^. In the next section, the Sumi-Marcus model and the approach to address ergodicity breaking are reviewed. The analytical formulation of our model is then presented. Key parameters used in the model are related to experimentally measurable quantities. Especially, a working model of photo-excited ET reactions is derived. Next, we discuss the predictions of the model in different classes of ET reactions and compare its results with those of the Sumi–Marcus model. We then apply the model to analyze the ET reactions in flavodoxin, which are representatives of ET reactions in the frozen and active regions. Built upon these theoretical and experimental results, the coupling mechanisms of ET reactions and environmental fluctuations in both equilibrium and nonequilibrium scenarios are comprehensively discussed. Limitations of current work and future directions are presented at the end.

## Results

### The Sumi–Marcus model

By treating the environmental fluctuations as a diffusive process^33^, Sumi and Marcus proposed a two-dimensional theory to model ET reactions^32^. A “fast” coordinate, denoted by *q*, was introduced to take into account the contributions from intramolecular vibrations, while a “slow” coordinate, denoted by *x*, was used to represent the local motions involved in a ET reaction. Mathematically, the ET dynamics is modeled as the time evolution of the distribution of reactants, *P*(*x*, *t*),

5 $$\frac{{\partial P\left( {x,t} \right)}}{{\partial t}} = {\cal{L}}P\left( {x,t} \right) - k\left( x \right)P\left( {x,t} \right),$$

where ${\cal{L}}$ is the Liouville operator that governs diffusion, and *k*(*x*) is the probability of reaction along the solvent coordinate, *x*. The reaction kernel *k*(*x*) is determined by the reaction barrier, being the intersection of free energy surfaces of the reactant and product states. As an immediate outcome of the theory, the reorganization energy *λ* is separated into two terms, the “inner” reorganization energy, *λ*_*i*_, coming from contributions of intramolecular vibrations, and the “outer” reorganization energy, *λ*_*o*_, from contributions of environmental fluctuations, i.e.,

6 $$\lambda = \lambda _i + \lambda _o.$$

The ET dynamics, measured as the survival probability of reactants, is obtained by integrating the reactants’ distribution, *P*(*x*, *t*),

7 $$Q(t) = \int_{ - \infty }^{ + \infty } {P\left( {x,t} \right){\mathrm{d}}x} .$$

The Sumi–Marcus model can produce ET dynamics with non-exponential behaviors and has been extensively applied to studying different kinds of ET reactions^4,14,15,34–36^. However, when the ET reaction is much faster than environmental relaxations (discussed as the “non-diffusing limit” in the work of Sumi and Marcus^32^), the model predicts a multi-exponential dynamics, in contrast to the single-exponential dynamics observed in a “frozen” environment^19^. On the other hand, the model assumed a fixed curvature of the free energy surface for the solvent coordinate. However, the curvature is proportional to thermal fluctuations, which is likely to be dependent on the ET reaction timescale because of ergodicity breaking of the system.

### The method of restricted ensembles

The broken of ergodicity for the system means that under certain constraints the complete phase space of the system is not accessible. In this work, it is the ET reaction that forbids parts of the phase space from being connected by slower relaxations as compared to the reaction. This phenomenon is known as the dynamical ergodicity breaking, because the breaking of ergodicity is not permanent and ergodicity can be recovered in longer timescales^37^. The physical properties of a nonergodic system have to be calculated by averages over restricted ensembles^38^.

One of the first applications of restricted ensembles in addressing ET reactions was made by Matyushov^39,40^. Assume that ET reaction rate is $k_{{\mathrm{ET}}} = 1/\left\langle {\tau _{{\mathrm{ET}}}} \right\rangle$, in which $\left\langle {\tau _{{\mathrm{ET}}}} \right\rangle$ is the average ET time constant, and the reaction coordinate *X* = *X*(*q*_1_, *q*_2_, …, *q*_*N*_) is a function of *N* independent coordinates {*q*_*i*_} of the system. The free energy of the system *F*(*X*), at the R or P state, is defined as

8 $$e^{-F\left( X \right)/k_BT} 	{\propto} \int {{\delta} \left( {X - X\left( {q_1, \ldots ,q_N} \right)} \right)e^{ - H\left( {q_1, \ldots ,q_N} \right)/k_BT}} \\ 	 \times \prod_{i,\left| {\omega} \right| < k_{ET}} {\delta} \left( {q_i\left( {\omega} \right) - Q_i} \right)\prod_{i,{\omega}} {{\mathrm{d}}q_i\left( \omega \right)},$$

in which *H*(*q*_1_, …*q*_*N*_) is the Hamiltonian of the system, and {*q*_*i*_(*ω*)} are the Fourier transformed coordinates. In contrast to the free energy defined in equilibrium, in which the density of state, exp(−*H*/*k*_*B*_*T*), is integrated over all possible values of *q*_*i*_, *i*=1, …, *N*, the above definition assumes motions that are slower than the ET reaction (|*ω*|<*k*_ET_) to be frozen and fixes the coordinate *q*_*i*_(*ω*) to be a constant *Q*_*i*_. As a result, *F*(*X*) is also dependent on the ET rate, *F*(*X*) = *F*(*X*, *k*_ET_). Although the restricted free energy function is not well defined in thermodynamics, in the later development, the free energy function can be used to quantify the magnitude of nonequilibrium local fluctuations within a finite time window.

### Nonergodic free energy surfaces

It is assumed that environmental fluctuations of a ET reaction can be approximately modeled as a polarization field which follows the Gaussian statistics^39^. The corresponding reaction coordinate, denoted by *x*, is defined as follows

9 $$x = - {\int} {{\mathrm{d}}\vec r\,\vec P\left( {\vec r} \right) \cdot {\mathrm{\Delta }}\vec E_0\left( {\vec r} \right),\quad {\mathrm{\Delta }}\vec E_0 = \vec E_1 - \vec E_2} ,$$

which is the polarization energy induced by the difference of two electric fields generated by the R (with the subscript 1) and P (with the subscript 2) states of the donor-acceptor pair and $\vec P\left( {\vec r} \right)$ is the polarization field of the solvent. According to restricted ensembles, the complete phase space is separated into a collection of isolated components. Within each component, the free energy curves along the coordinate *x* can be derived,

10a $$F_1\left( {x,k_{{\mathrm{ET}}}} \right) = \frac{{x^2}}{{4\lambda _o\left( {k_{{\mathrm{ET}}}} \right)}},$$

10b $$F_2\left( {x,k_{{\mathrm{ET}}}} \right) = \frac{{\left( {x - 2\lambda _o\left( {k_{{\mathrm{ET}}}} \right)} \right)^2}}{{4\lambda _o\left( {k_{{\mathrm{ET}}}} \right)}} + {\mathrm{\Delta }}G_{{\mathrm{sol}}}\left( {k_{{\mathrm{ET}}}} \right),$$

in which all parameters are dependent on the reaction rate, *k*_ET_^39^. In these expressions, the minimum of the R state’s free energy, *F*_1_(*x*, *k*_ET_), is shifted to the origin. Δ*G*_sol_(*k*_ET_) is the nonergodic reaction free energy, contributed by active relaxation modes of the environment. *λ*_*o*_(*k*_ET_) is the nonergodic outer reorganization energy, defined by^39^,

11 $$\lambda _o\left( {k_{{\mathrm{ET}}}} \right) = \lambda _o^{eq}{\int}_{ - \infty }^{ + \infty } {{\mathrm{d}}\omega S\left( \omega \right)\theta \left( {\left| \omega \right| - k_{{\mathrm{ET}}}} \right)} ,$$

where *θ*(*x*) is the Heaviside step function and

12 $$\lambda _o^{{\mathrm{eq}}} = \mathop {{{\mathrm{lim}}}}\limits_{k_{{\mathrm{ET}}} \to 0} \lambda _o\left( {k_{{\mathrm{ET}}}} \right),$$

which is the reorganization energy when all environmental relaxation modes are fully relaxed. Here *S*(*ω*)^41^,

13 $$S\left( \omega \right) = \frac{1}{\pi }{\int}_0^\infty {{\mathrm{d}}t\,S\left( t \right){\mathrm{cos}}\omega t} ,$$

is the Fourier transform of the time auto-correlation function (TCF) of the polarization energy *x*, *S*(*t*),

14 $$S\left( t \right) = \frac{{\left\langle {\delta x\left( t \right)\delta x\left( 0 \right)} \right\rangle }}{{\left\langle {\delta ^2x\left( 0 \right)} \right\rangle }},$$

where $\delta x\left( t \right) = x\left( t \right) - \left\langle x \right\rangle _{t \to \infty }$. The stabilization energy, which is measurable in the solvation experiment^16^, is defined as

15 $${\mathrm{\Delta }}E_{{\mathrm{sol}}} = \left\langle x \right\rangle _{t = 0} - \left\langle x \right\rangle _{t \to \infty }.$$

As a result, a dynamic factor, denoted by *γ*, can be defined to quantify the interplay between the ET reaction and environmental fluctuations,

16 $$\gamma = \int_{ - \infty }^{ + \infty } {{\mathrm{d}}\omega S\left( \omega \right)\theta \left( {\left| \omega \right| - k_{{\mathrm{ET}}}} \right)} .$$

Therefore, *γ* is a function of *k*_ET_. By definition, *λ*_*o*_(*k*_ET_) and Δ*G*_sol_(*k*_ET_) are related to their equilibrium values through (see Supplementary Eq. 10)

17 $$\lambda _o^\gamma = \lambda _o\left( {k_{{\mathrm{ET}}}} \right) = \gamma \lambda _o^{{\mathrm{eq}}},$$

18 $${\mathrm{\Delta }}G_{{\mathrm{sol}}}^\gamma = {\mathrm{\Delta }}G_{{\mathrm{sol}}}\left( {k_{{\mathrm{ET}}}} \right) = {\mathrm{\Delta }}G_{{\mathrm{sol}}}^{{\mathrm{eq}}} + \left( {1 - \gamma } \right)\lambda _o^{{\mathrm{eq}}}.$$

It turns out that all the correction of nonergodicity can be included into *γ*. To simplify the notations in the following discussion, a superscript of *γ* is labeled onto a parameter if it is dependent on the ET rate, *k*_ET_, such as $\lambda _o^\gamma$ and ${\mathrm{\Delta }}G_{{\mathrm{sol}}}^\gamma$.

The *γ* values vary between 0 and 1 (see Fig. 1b). When the ET reaction is fast relative to motions of the environment, *γ*→0, few environmental motions are able to couple with the reaction. The local fluctuations are limited within a fraction of the complete phase space, which results in a steep free energy surface along the solvent coordinate *x*, hence a small *λ*_*o*_(*k*_ET_) (see the blue curve in Fig. 1c). When the ET is slow, *γ*→1, the system is able to sample a majority of the environment’s phase space, leading to a flat free energy surface and a large $\lambda _o^\gamma$ (see the red curve in Fig. 1c).

In the above discussion, the donor-acceptor pair is simplified to be a structure-less dipole. However, in general, the degrees of freedom inside the donor and acceptor are also relevant to the ET reaction. Following the argument of Sumi and Marcus^32^, it is assumed that these intramolecular degrees of freedom, governed by the Gaussian statistics, respond to any change of the system instantly. Another coordinate, denoted by *q*, is used to represent these degrees of freedom. Hence, the free energy surfaces, as a function of *x* and *q*, are expressed as

19a $$F_1^\gamma \left( {x,q} \right) = \frac{{x^2}}{{4\lambda _o^\gamma }} + \frac{{q^2}}{{4\lambda _i}},$$

19b $$F_2^\gamma \left( {x,q} \right) = \frac{{\left( {x - 2\lambda _o^\gamma } \right)^2}}{{4\lambda _o^\gamma }} + \frac{{\left( {q - 2\lambda _i} \right)^2}}{{4\lambda _i}} + {\mathrm{\Delta }}G^\gamma ,$$

where *λ*_*i*_ is the inner reorganization energy contributed by intramolecular vibrations. Δ*G*^*γ*^ is the free energy of the ET reaction, including contributions from both the intramolecular degrees of freedom and active environmental relaxation modes. It is related to its equilibrium value through

20 $${\mathrm{\Delta }}G^\gamma = {\mathrm{\Delta }}G^o + \left( {1 - \gamma } \right)\lambda _o^{eq}.$$

In Fig. 1d, the energy surface on top of the figure corresponds to a fast ET reaction relative to environmental motions, whose intersection along *x* is the blue curve in Fig. 1c. The flattest energy surface corresponds to a slow ET, whose intersection along *x* is the red curve in Fig. 1c. The gradual change of Δ*G*^*γ*^ with respect to the relative rates between ET and environmental fluctuations can be seen in both figures.

### The equation of motion for the system

The reaction kernel *k*(*x*) is determined by the intersection of the two free energy surfaces,

21 $$k\left( x \right) \propto {\mathrm{exp}}\left( { - \frac{1}{{k_BT}}\left[ {F_1^\gamma \left( {x,q^\dagger \left( x \right)} \right) - F_1^\gamma \left( {x,0} \right)} \right]} \right),$$

where the transition state *q*^†^(*x*) at each point of *x* is determined by the following equation,

22 $$F_1^\gamma \left( {x,q^\dagger \left( x \right)} \right) = F_2^\gamma \left( {x,q^\dagger \left( x \right)} \right).$$

In the nonadiabatic limit, *k*(*x*) is given by the following equation^32^,

23 $$k\left( x \right) = \frac{{J^2}}{\hbar }\sqrt {\frac{\pi }{{\lambda _ik_BT}}} e^{ - \frac{{\left( {x - \left( {{\mathrm{\Delta }}G^\gamma + \lambda ^\gamma } \right)} \right)^2}}{{4k_BT\lambda _i}}},$$

where *λ*^*γ*^ is the total reorganization energy, subject to the nonergodic correction, and $\lambda ^\gamma = \lambda _i + \lambda _o^\gamma$. The electronic coupling *J* is assumed to be independent of the configurations of the environment, given that the donor and acceptor are close in distance.

On the other hand, assuming that environmental fluctuations can be modeled by a diffusive process, in the overdamped limit, a Liouville operator similar to the Sumi–Marcus theory, is derived^42^ (see Supplementary Eq. 29)

24 $${\cal{L}}P = D\left( t \right)\left\{ {2\lambda _o^\gamma k_BT\frac{{\partial ^2}}{{\partial x^2}}P + x\frac{\partial }{{\partial x}}P + P} \right\},$$

where *D*(*t*) is the diffusion coefficient that satisfies

25 $$D\left( t \right) = - \frac{1}{{S\left( t \right)}}\frac{{{\mathrm{d}}S\left( t \right)}}{{{\mathrm{d}}t}},$$

in which *S*(*t*) is the normalized TCF of the solvation dynamics defined in Eq. 14. Thus, the time evolution of the reactant’s distribution is determined by

26 $$\frac{\partial }{{\partial t}}P = D\left( t \right)\left\{ {2\lambda _o^\gamma k_BT\frac{{\partial ^2}}{{\partial x^2}}P + x\frac{\partial }{{\partial x}}P + P} \right\} - k\left( x \right)P.$$

### Photo-excited ET reactions

In the case of a photo-excited ET reaction, upon excitation, the local environment of the donor and acceptor is close to the global minimum of the ground state, deviating slightly from that of the excited state, which is the R state of the ET reaction^43^. This deviation along the coordinate *x* is denoted by Δ*x*^*γ*^, which has the relation with its equilibrium value, Δ*x*^*γ*^ = Δ*x*^eq^ × *γ*. Since Δ*x*^eq^ is approximately equal to Δ*E*_sol_, the stabilization energy (see Eq. 15), we have (see Supplementary Eq. 32),

27 $${\mathrm{\Delta }}x^\gamma = \gamma {\mathrm{\Delta }}E_{{\mathrm{sol}}}.$$

On the other hand, the reaction free energy Δ*G*^*γ*^ also differs slightly from Eq. 20 (see Supplementary Eq. 34), i.e.,

28 $${\mathrm{\Delta }}G^\gamma = {\mathrm{\Delta }}G^o + \left( {1 - \gamma } \right)\lambda _o^{{\mathrm{eq}}} + \left( {1 - \gamma } \right){\mathrm{\Delta }}E_{{\mathrm{sol}}}.$$

To conclude this section, in a photo-excited ET reaction, the time evolution of the reactants’ population, *P* (*x*, *t*), is governed by the equation

29 $$\frac{\partial }{{\partial t}}P = D\left( t \right)\left\{ {2\lambda _o^\gamma k_BT\frac{{\partial ^2}}{{\partial x^2}}P + \left( {x - \gamma {\mathrm{\Delta }}E_{{\mathrm{sol}}}} \right)\frac{\partial }{{\partial x}}P + P} \right\} - k\left( x \right)P,$$

where *k*(*x*) and the initial condition of *P*(*x*, *t*) are given by (see Supplementary Eq. 35)

$$k\left( x \right) = \frac{{J^2}}{\hbar }\sqrt {\frac{\pi }{{\lambda _ik_BT}}} {\mathrm{exp}}\left[ { - \frac{{\left( {x - \gamma {\mathrm{\Delta }}E_{{\mathrm{sol}}} - \left( {{\mathrm{\Delta }}G^\gamma + \lambda ^\gamma } \right)} \right)^2}}{{4k_BT\lambda _i}}} \right],$$

30 $$P\left( {x,t = 0} \right) = \sqrt {\frac{1}{{4\pi \lambda _o^\gamma k_BT}}} {\mathrm{exp}}\left[ { - \frac{{x^2}}{{4\lambda _o^\gamma k_BT}}} \right].$$

In the above expressions, for convenience, the center of the initial distribution, *P* (*x*, *t* = 0), is shifted to *x* = 0.

Finally, if the solvation TCF can be expressed as a multi-exponential decay, which is $S\left( t \right) = \mathop {\sum}\nolimits_i {c_ie^{ - t/\tau _i}}$, the dynamic factor can be evaluated by (see Supplementary Eq. 38)

31 $$\gamma = 1 - \frac{2}{\pi }\mathop {\sum}\nolimits_i {c_i{\mathrm{arctan}}\left[ {\tau _i/\left\langle {\tau _{{\mathrm{ET}}}} \right\rangle } \right]} .$$

### Effects of environmental relaxations on ET dynamics

As it is seen in Eq. 29, the coupling between the ET reaction and environmental fluctuations is reflected in the Liouville operator ${\cal{L}}$ (Eq. 24) and the modified parameters, such as $\lambda _o^\gamma$ and Δ*G*^*γ*^, which are revised by the dynamic factor, *γ* (Eq. 16). In this section, this coupling mechanism is elucidated. Furthermore, although it has been well known that the solvation dynamics in biological systems usually shows non-Debye behaviors, its effects on the ET dynamics are not clearly understood. The following discussion will try to shed some light on this problem by examining the influence of solvation dynamics with single-exponential and double-exponential decays. In this discussion, the ET dynamics, *Q*(*t*) (Eq. 7), is fitted with stretched-exponential decays (Eq. 4), in which the average ET time constant is given by

32 $$\left\langle {\tau _{{\mathrm{ET}}}} \right\rangle = \tau _{{\mathrm{ET}}}\frac{{{\mathrm{\Gamma }}\left( {1/\beta } \right)}}{\beta },$$

where Γ(*z*) is the standard Gamma function.

### Debye relaxations

**Fig. 2:**
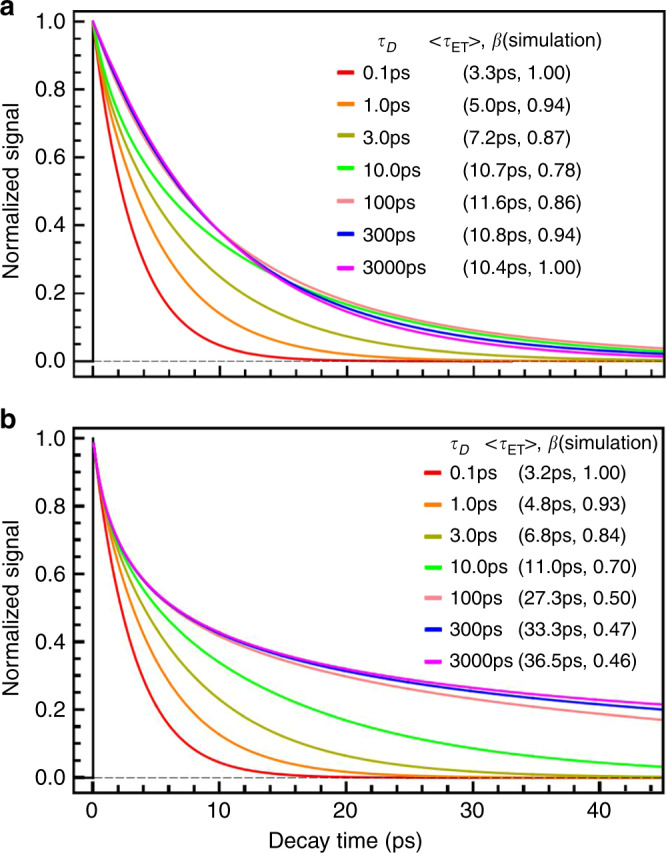
Simulations of photo-excited ET dynamics. The simulations are performed with different solvation timescales, *τ*_*D*_, while fixing other parameters at *J* = 0.020 eV, Δ*G*^*o*^ = −0.60 eV, *λ*_*i*_ = 0.80 eV, and *λ*_*o*_ = 0.40 eV. It is assumed that excited-state (ES) global minimum is aligned with the ground state (GS) global minimum along the *x* coordinate. **a** Simulations using the nonergodic model. **b** Simulations using the Sumi-Marcus model. Note the difference in the variation of *β* between two models.

**Fig. 3:**
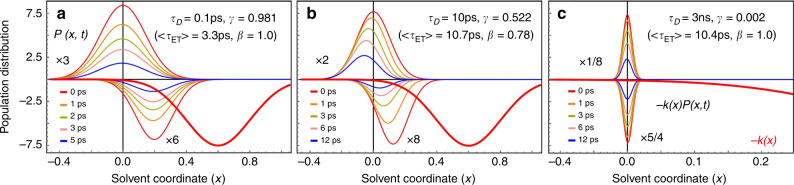
Simulations of time evolution of reactants’ distribution, *P*(*x*, *t*). The simulations are performed with different solvation timescales *τ*_*D*_, while  values of other parameters used are the same as those in Fig. 2. The bold red line represents the curve of −*k*(*x*) without scaling. Within each graph, the upper panel displays the time evolution of *P*(*x*, *t*), while the lower panel displays the evolution of the reaction rate’s distribution, −*k*(*x*)*P*(*x*, *t*). **a** *τ*_*D*_ = 0.1 ps. **b** *τ*_*D*_ = 10 ps. **c** *τ*_*D*_ = 3 ns.

With the Debye relaxation, the TCF of the solvent coordinate *x* is a single-exponential decay,

33 $$S\left( t \right) = e^{ - t/\tau _D},$$

which leads to a constant diffusion coefficient *D*(*t*) = 1/*τ*_*D*_. To find out how *τ*_*D*_ affects the ET dynamics, it is helpful to compare the solutions of Eq. 29 with different values of *τ*_*D*_ while fixing all other parameters in the equation, as shown in Fig. 2. It is clear that with the increase of *τ*_*D*_, *β* decreases from 1.0 and then increases toward 1.0 after reaching a minimum (Fig. 2a). In comparison, the ET dynamics modeled by the Sumi–Marcus model displays very different behaviors (Fig. 2b). The stretched factor *β* monotonically decreases with the increase of *τ*_*D*_. To understand these different dynamics, it is worthwhile examining the time evolution of the reactants’ distribution, *P*(*x*, *t*) (Fig. 3). In the region where $\tau _D \ll \left\langle {\tau _{{\mathrm{ET}}}} \right\rangle$, local motions quickly bring *P*(*x*, *t*) back to equilibrium (Fig. 3a). The ET dynamics fits an exponential decay with its reaction rate well predicted by the Marcus theory (Eq. 2). This result is also consistent with the simulation of the Sumi–Marcus model (see Supplementary Fig. 1a). The ET reaction belongs to the equilibrium class with $\lambda _o^\gamma$ and ${\mathrm{\Delta }}G_{{\mathrm{sol}}}^\gamma$ equal to their equilibrium values, i.e., *γ* → 1 (Fig. 1 Class III). As *τ*_*D*_ becomes larger, the ET reaction gets into the active region, where $\tau _D\sim \left\langle {\tau _{{\mathrm{ET}}}} \right\rangle$ (Fig. 1 Class II), and *γ* → 1. $\lambda _o^\gamma$ and ${\mathrm{\Delta }}G_{{\mathrm{sol}}}^\gamma$ deviate from their equilibrium values. The ET dynamics is stretched because *P*(*x*, *t*) is driven away from its equilibrium distribution by the asymmetric distribution of the reaction kernel, *k*(*x*), with respect to *x* = 0 (Fig. 3b). In the other limit, where *τ*_*D*_ ≫ *τ*_ET_, the environment is frozen (Fig. 1 Class I). The ET dynamics simulated by the Sumi-Marcus model is very stretched, which is the result of the static heterogeneity of the environment (see Supplementary Fig. 1c). However, on the other hand, the ET dynamics simulated by current model is exponential. In this limit, the ET reaction happens with negligible impacts from local fluctuations, *γ* → 0. Mathematically, the static heterogeneity has the same effects on $\lambda _o^\gamma$ as well as Δ*G*^*γ*^, which cancel out and result in the exponential dynamics. The reaction rate is mostly determined by the value of *k*(*x*) at *x* = 0 (Fig. 3c). In this case, $\lambda _o^\gamma \to 0$, and ${\mathrm{\Delta }}G^\gamma \to {\mathrm{\Delta }}G^o + \lambda _o^{{\mathrm{eq}}}$. Additionally, the introduction of a non-zero stabilization energy (Eq. 15) modifies the ET dynamics slightly (see Supplementary Fig. 2 and 3).

### Non-Debye relaxations

**Fig. 4:**
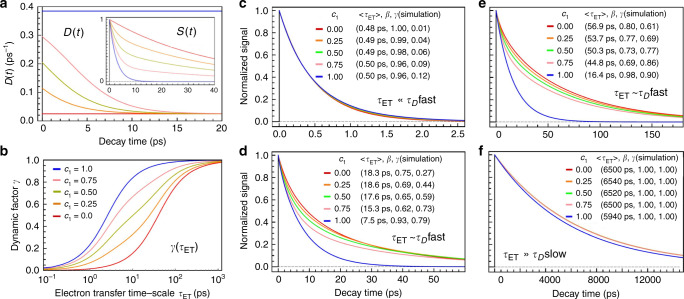
Simulations of ET dynamics with bi-exponential solvation process. The solvation correlation function is given by, $S\left( t \right) = c_1e^{ - t/\tau _D^{{\mathrm{fast}}}} + \left( {1 - c_1} \right)e^{ - t/\tau _D^{{\mathrm{slow}}}}$, where $\tau _D^{{\mathrm{fast}}} = 2.6$ ps, and $\tau _D^{{\mathrm{slow}}} = 40$ ps. **a** Evolution of the diffusion coefficient, *D*(*t*) (Eq. 25), with different values of *c*_1_. The inset is the evolution of *S*(*t*) (Eq. 14). **b** Evolution of the dynamic factor *γ* as a function of *τ*_*ET*_ with different values of *c*_1_. **c** Simulations of ET dynamics with different values of *c*_1_ while $\tau _{{\mathrm{ET}}} \ll \tau _D^{{\mathrm{fast}}}$. The values of parameters used are, *J* = 0.02 eV, Δ*G*^*o*^ = −0.9 eV, *λ*_*i*_ = 0.8 eV, $\lambda _o^{{\mathrm{eq}}} = 0.4$ eV, and Δ*E*_sol_ = 0.026 eV. **d** Simulations of ET dynamics with different values of *c*_1_ while $\tau _{{\mathrm{ET}}}\sim \tau _D^{{\mathrm{fast}}}$. Here, Δ*G*^*o*^ = −0.6 eV, and other parameters are the same as **c**. **e** Simulations of ET dynamics with different values of *c*_1_ while $\tau _{{\mathrm{ET}}}\sim \tau _D^{{\mathrm{slow}}}$. Here, Δ*G*^*o*^ = −0.5 eV. **f** Simulations of ET dynamics with different values of *c*_1_ while $\tau _{{\mathrm{ET}}} \gg \tau _D^{{\mathrm{slow}}}$. Here, Δ*G*^*o*^ = −0.2 eV.

The picture gets more intricate when moving beyond the Debye relaxation. To explain the influence of a solvation dynamics with multiple components on ET reactions, a solvation TCF with a double-exponential decay is used in Fig. 4,

34 $$S\left( t \right) = c_1e^{ - t/2.6} + \left( {1 - c_1} \right)e^{ - t/40}.$$

The time constants in picoseconds are chosen from the measured solvation dynamics of flavodoxin^18^, but the overall picture is applicable to biological ET systems in general. The solvation dynamics with a time component of a few picoseconds and another of tens of picoseconds have been observed in a variety of protein-water systems^16,44–47^. Different types of ET dynamics are produced by varying the coefficient *c*_1_ in *S*(*t*) in different sets of other parameters, in order to simulate ET dynamics in equilibrium, active and frozen environments (Fig. 1b). At any given time *t* > 0, the diffusion coefficient *D*(*t*) monotonically decreases with decreasing *c*_1_, suggesting a slowdown of local fluctuations (Fig. 4a). Similarly, at any given *τ*_ET_, the dynamic factor *γ* decreases with decreasing *c*_1_ (Fig. 4b). Since *D*(*t*) decays to the slower relaxation rate and *γ* gets close to 1 when *τ*_ET_→∞, it is reasonable to infer that the variation of *D*(*t*) is only relevant to the ET dynamics with *τ*_ET_ comparable to either of the relaxation timescales. As it is expected, in the frozen environment (Fig. 1b I), varying *S*(*t*) does not have a significant impact on the resulting ET dynamics (Fig. 4c). This also applies to the equilibrium class (Fig. 1b III). As long as the ET reaction is much slower than any local motion of the environment, the ET dynamics stays close to the prediction of the Marcus theory (Fig. 4f). However, inside the active region (Fig. 1b II), the ET dynamics is very sensitive to the coefficient of each solvation component, especially that of the slow component (Fig. 4d, e).

In summary, the determining factor of ET dynamics is the relative ratio between the solvation timescale and the ET timescale, which is quantified by a dynamic factor, *γ* (Eq. 16). The coupling mechanism between ET and the solvation is realized through the diffusion process and modification of thermodynamic properties, Δ*G*^*γ*^ and *λ*^*γ*^ (Eqs. 17 and 20). In the case where the solvation is much slower than ET, a small number of local relaxation modes are coupled to the reaction and the environment is frozen. This results in a single-exponential ET dynamics in which the complexity of the solvation dynamics is irrelevant. While the solvation is much faster, all local motions are coupled to ET and the environment is in equilibrium, which also leads to a single-exponential decay. It is when the solvation has a comparable timescale with that of the ET reaction, the dynamics becomes complicated. All components of the solvation dynamics should be taken into account to produce a reasonable picture of the ET reaction. Furthermore, the stabilization energy, which comes from the misalignment of the ground state and the excited state of the donor-acceptor pair, is also needed to reproduce an accurate ET dynamics.

### Analysis of ET reactions in flavodoxin

In this section, the model (Eq. 29) is applied to analyzing two types of photo-excited ET reactions in flavodoxin^15,19^. As discussed, to get an accurate picture of the ET reaction, detailed information of the solvation dynamics is required. The ET reactions to be analyzed are between a photo-excited flavin cofactor (FMN^*^ or FMNH^•*^) and a nearby tryptophan residue in flavodoxin. The TCF of their solvation dynamics is in the form of

35 $$S\left( t \right) = c_1e^{ - t/\tau _1} + c_2e^{ - t/\tau _2} + c_3e^{ - t/\tau _3},$$

with *τ*_1_ being a few ps, *τ*_2_ being tens of ps, and *τ*_3_ being hundreds of ps^16,18^. The solvation dynamics does not have sub-picosecond components, which are attributed to ballistic motions of bulk water, because the functional site, in which the solvation is measured, is buried deeply inside the binding pocket and is distant from the bulk water. The experimental solvation dynamics of flavodoxin with FMNH^•^ does not have a third component due to the shorter lifetime of FMNH^•*^^18^. Within each system, mutants are chosen such that each mutant is mutated at the same site and does not drastically change the protein’s conformation, and the structures of the donor and acceptor, it is therefore assumed that the solvation TCF, *S*(*t*), and the inner reorganization energy, *λ*_*i*_, stay invariant under mutation. Given that the equilibrium value of each mutant’s reaction free energy, Δ*G*^*o*^, is available, the reorganization energies, $\lambda _o^\gamma$ and *λ*_*i*_, can be obtained by fitting the experimental ET dynamics with Eq. 29.

**Fig. 5:**
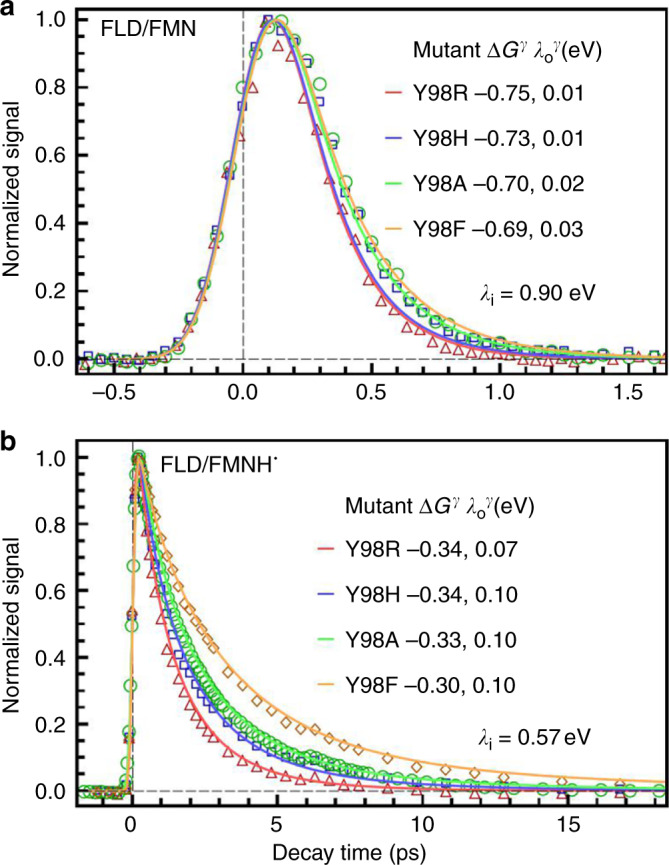
Simulations of experimental ET dynamics in flavodoxin. Simulations (solid lines) for different mutants are compared with experimental results (symbols). All time constants are in the unit of ps. **a** The ET reaction in flavodoxin/FMN (oxidized state) with *S*(*t*) = 0.53*e*^−*t*/1.0^ + 0.26*e*^−*t*/25^ + 0.21*e*^−*t*/670^. **b** The ET reaction in flavodoxin/FMNH^•^ (semiquinone) with *S*(*t*) = 0.76*e*−*t*/2.6 + 0.24*e*^−*t*/40^.

The ET reaction of FMN is in the frozen region (Fig. 1b I) and displays a single-exponential dynamics with the reaction rate being in the hundreds of fs. As expected, the outer reorganization energy for each mutant is close to 0, a characteristic of ET reactions in the frozen region (Table 1 and Fig. 5a). The large reaction rate is a result of low activation energy, being determined by the large driving force, |Δ*G*^*γ*^|, as well as a fairly large inner reorganization energy, *λ*_*i*_. It has been suggested that the unusual value of *λ*_*i*_ comes from the significant structural change between FMN and the negatively charged, FMN^−^^48^. On the other hand, the ET dynamics with FMNH^•^, being in the active region (Fig. 1b II), is stretched with its reaction timescale comparable to the shortest solvation timescale, *τ*_1_. The model gives a small but nontrivial $\lambda _o^\gamma$ (Table 1 and Fig. 5b). From Table 1, it is obvious that the calculated reaction free energy Δ*G*^*γ*^ deviates significantly from its equilibrium value for each mutant.

**Table 1:** Energetics of forward electron transfer reactions in flavodoxin^a^.

	Mutants	$\left\langle {\tau _{{\mathrm{ET}}}} \right\rangle$/ps	*β*	*γ*	*λ*_*i*_	$\lambda _o^\gamma$	*λ*^*γ*^	Δ*G*^*γ*^	Δ*G*^*o*^
Flavodoxin (FMN)b	Y98R	0.19	1.0	0.07	0.90	0.01	0.91	−0.75	−0.97
	Y98H	0.20	1.0	0.07	0.90	0.01	0.91	−0.73	−0.96
	Y98A	0.25	1.0	0.08	0.90	0.02	0.92	−0.70	−0.95
	Y98F	0.26	1.0	0.09	0.90	0.03	0.93	−0.69	−0.99
Flavodoxin (FMNH^•^)c	Y98R	1.4	0.83	0.24	0.57	0.07	0.64	−0.34	−0.59
	Y98H	1.8	0.76	0.30	0.57	0.10	0.67	−0.35	−0.59
	Y98A	2.2	0.79	0.35	0.57	0.10	0.67	−0.34	−0.55
	Y98F	3.4	0.83	0.45	0.57	0.10	0.67	−0.30	−0.44

^a^All energies are in the unit of eV. $\left\langle {\tau _{{\mathrm{ET}}}} \right\rangle$ and *β* are obtained by directly fitting experimental data with the stretched-exponential function; The ET dynamics of the oxidized state is represented by the fluorescence transients of FMN^*^ in different mutants gated at 538 nm and that of the semiquinone state is represented by the absorption transients of FMNH^•*^ in different mutants probed at 800 nm; *γ* is computed using Eq. 31; *λ*_*i*_, assumed to be invariant under mutation, and $\lambda _o^\gamma$ are obtained by fitting experimental data with Eq. 29; *λ*^*γ*^ is the sum of *λ*_*i*_ and $\lambda _o^\gamma$; Δ*G*^*γ*^ are computed using Eq. 28; Δ*G*^*o*^ for different mutants are obtained from literature^15,19^.

^b^For Flavodoxin/FMN, *J* = 20 meV^19^.

^c^For Flavodoxin/FMNH^•^, *J* = 14 meV^15^.

In contrast to the predictions made by the Sumi–Marcus model (Fig. 2b and Supplementary Fig. 1), the lack of strong dependencies on the static heterogeneity of the environment in experimental results suggests that, for ultrafast short-range ET reactions, the electronic and vibrational structures of the donor and acceptor are insensitive to the fluctuations of the local environment. The influences of environmental fluctuations on the ET dynamics are reflected in their contributions to the outer reorganization energy as well as the driving force of the reaction. Conversely, if the ET dynamics in the frozen region displays non-exponential decay, it might suggest that the donor and acceptor are strongly coupled with the environment such that the configurations of the surrounding molecules have an impact on the electronic and vibrational structures of the donor and acceptor^49^.

## Discussion

To conclude, in this work, a model of ultrafast short-range electron transfer reactions is proposed to address the nonequilibrium interactions between ET reactions and environmental fluctuations. When dealing with ET reactions in equilibrium (Fig. 1b III), it is shown that the new model is consistent with the classic Marcus theory (Eq. 2). In the case of non-equilibrium ET reactions (Fig. 1b I and II), this model predicts a nonergodic ET dynamics by freezing relaxation modes that are slower than ET reactions. This model successfully explained the single-exponential behavior of extremely fast ET between FMN and the tryptophan (tyrosine) residue in flavodoxin (Fig. 1b I), which does not fit into the classic physical picture. Because of the existence of frozen relaxation modes, the reaction free energy and the reorganization energy of nonequilibrium ET reactions can deviate significantly from their values at equilibrium. Particularly, the ET reaction in the frozen region is characterized by $\lambda _o^\gamma \approx 0$. This model improves our current understanding of ultrafast (short-range) ET reactions, which is instrumental to our comprehension of physical mechanisms behind many biological functions.

It should be noted that there still exist a few limitations in current work. Firstly, when applying the model to analyze experimental results, the relevant parameters, such as the inner and outer reorganization energy, are obtained by fitting the simulated results with experimental data. This approach certainly has its limitation, especially when more parameters like Δ*G* and the solvation correlation function *S*(*t*) are not available experimentally. Therefore, the applicability of the model can be enhanced if it is integrated with ab initio methods, which can help to compute these model parameters. Secondly, this approach of addressing photo-excited ET assumes that vibrational distributions of the reactant and product states of the reaction follow the Boltzmann distribution. However, it has been argued that photo-excited ET reactions involve vibrationally excited states, whose relaxation is in the range of a few picoseconds^15^. Therefore, the phenomenon of nonequilibrium also exists inside the donor-acceptor pair. In this case, instead of a two-state model for the donor and acceptor, a fine model with electronic and vibrational structures is required for a reasonable description^49^. This further complicates the picture and more efforts are on the way.

We also want to emphasize that, due to the lack of theoretical tools, it is very likely that many reactions that have been discovered in the past could belong to ET reactions in the frozen region while stay unnoticed. Furthermore, given the universal existence of ergodicity breaking in complex environments^37,50^, this new model can be applied to any ultrafast electron-transfer reactions with the coupling between ET systems and their environments, although it was initially developed for treatment of photo-excited ET dynamics in biology. It should find wide applications in chemistry, materials, and biology, to gain a deep understanding of the fundamental ultrafast ET processes in those fields.

## Methods

### Computational routine for solving the differential equation (Eq. (29))

In this work, we used the software called Mathematica to solve the differential equations. The software provides numerical methods for solving ordinary or partial differential equations, such as NDSolve, which are simple to use. The solution of NDSolve is then numerically integrated using methods, such as NIntegrate, to give *Q*(*t*) (Eq. (4)). *Q*(*t*) can be fitted by a stretched-exponential function using the method, NonlinearModelFit.

## Supplementary information

Supplementary Information

Peer Review

## Data Availability

The data that support the findings of this study are available from the corresponding author (D.Z.) upon reasonable request.

## Code availability

The Mathematica notebook for solving the differential equations is uploaded as the Supplementary File, *et_code.nb*.
