# Supplementary material for: Effects of nonequilibrium fluctuations on ultrafast short-range electron transfer dynamics
Source: Nat Commun. 2020 Jun 4;11:2822. doi: 10.1038/s41467-020-15535-y (PMC7272615; doi:10.1038/s41467-020-15535-y)
Supplement: Supplementary file 1 — Supplementary Information [file 41467_2020_15535_MOESM1_ESM.pdf]

Effects of nonequilibrium fluctuations on ultrafast short-range electron transfer dynamics

Supplementary Information

Lu et al.

Supplementary Information for Effects of nonequilibrium fluctuations on ultrafast short-range electron transfer dynamics

Yangyi Lu[1], Mainak Kundu[1] & Dongping Zhong[1,2]

[1]*Department of Physics, Department of Chemistry and Biochemistry, Programs of Biophysics, Chemical Physics, and Biochemistry, The Ohio State University, Columbus, Ohio 43210, United States.*
[2]*Center for Ultrafast Science and Technology, School of Chemistry and Chemical Engineering, School of Physics and Astronomy, Shanghai Jiao Tong University, Shanghai 200240, China.*
(email: zhong.28@osu.edu)

This file of supplementary information contains detailed derivations of key equations used in the main text.

Supplementary Note 1: The nonergodic reaction free energy

We start with the theoretical framework used by Matyushov[1]. The solvent coordinate, x, is defined as the polarization energy induced by the difference dipole between the dipoles generated by the reactant (R) and product (P) states of the ET reaction,

$$x = -\int \mathrm{d}\vec{r}\, \vec{P}(\vec{r}) \cdot \Delta\vec{E}_0(\vec{r}), \quad \Delta\vec{E}_0 = \vec{E}_1 - \vec{E}_2, \tag{1}$$

where $\vec{E}_1$ and $\vec{E}_2$ are electric fields generated by the R and P states of the donor-acceptor pair. Assume that the polarization field $\vec{P}(\vec{r})$ is linearly proportional to the electric field,

$$\vec{P}(\vec{r}) = \int \mathrm{d}\omega \int \mathrm{d}\vec{r}'\, \chi(\omega, \vec{r}, \vec{r}') \cdot \vec{E}(\vec{r}'), \tag{2}$$

where $\chi(\omega, \vec{r}, \vec{r}')$ is the dielectric tensor which is dependent on the frequency ω. It is assumed that the R state of the system is in equilibrium with the environment. That is, at $t = 0$, the system is "frozen" at the polarization[1],

$$\vec{P}_f(\vec{r}) = \int \mathrm{d}\omega \int \mathrm{d}\vec{r}'\, \chi(\omega, \vec{r}, \vec{r}') \cdot \vec{E}_1(\vec{r}'). \tag{3}$$

Hence, we define the origin of the solvent coordinate to be

$$x_0 = -\int \mathrm{d}\vec{r}\, \vec{P}_f(\vec{r}) \cdot \Delta\vec{E}_0(\vec{r}), \quad \Delta\vec{E}_0 = \vec{E}_1 - \vec{E}_2, \tag{4}$$

Equivalently, the solvent coordinate is redefined to be

$$x = -\int \mathrm{d}\vec{r}\, (\vec{P}(\vec{r}) - \vec{P}_f(\vec{r})) \cdot \Delta\vec{E}_0(\vec{r}). \tag{5}$$

Using the method of restricted ensembles[2], we let the cutoff frequency of the environmental relaxation modes be the average ET rate k_{ET}. The *nonergodic* solvent coordinate with a given k_{ET} is written by

$$x = -\int \mathrm{d}\omega\, \theta(|\omega| - k_{ET}) \int \mathrm{d}\vec{r}_1 \int \mathrm{d}\vec{r}_2\, (\vec{E}(\vec{r}_1) - \vec{E}_1(\vec{r}_1)) \cdot \chi(\omega, \vec{r}_1, \vec{r}_2) \cdot \Delta\vec{E}_0(\vec{r}_2). \tag{6}$$

The physical picture behind this method is that only relaxation modes that are faster than the cutoff frequency, k_{ET}, actively contribute to the polarization of the system, while all the slow modes stay in equilibrium with $\vec{E}_1$, the R state of the system. Then, the difference of polarization energies of the R and P states, ΔG_{sol}, is given by[1]

$$\Delta G_{sol}(k_{ET}) = \int \mathrm{d}\omega\theta(|\omega| - k_{ET}) \int \mathrm{d}\vec{r}_1 \int \mathrm{d}\vec{r}_2 \Big(\frac{1}{2}\vec{E}_1(\vec{r}_1) \cdot \chi(\omega, \vec{r}_1, \vec{r}_2) \cdot \vec{E}_1(\vec{r}_2) - \frac{1}{2}\vec{E}_2(\vec{r}_1) \cdot \chi(\omega, \vec{r}_1, \vec{r}_2) \cdot \vec{E}_2(\vec{r}_2)\Big), \tag{7}$$

and the outer reorganization energy λ_o, including contributions of the environmental relaxation modes, reads

$$\lambda_o(k_{ET}) = \int \mathrm{d}\omega\theta(|\omega| - k_{ET}) \int \mathrm{d}\vec{r}_1 \int \mathrm{d}\vec{r}_2 \, \frac{1}{2}\Delta\vec{E}_0(\vec{r}_1) \cdot \chi(\omega, \vec{r}_1, \vec{r}_2) \cdot \Delta\vec{E}_0(\vec{r}_2). \tag{8}$$

Both are dependent on the reaction rate k_{ET}. It is easily seen that

$$\begin{aligned}\Delta G_{sol}(k_{ET}) + \lambda_o(k_{ET}) &= \int \mathrm{d}\omega\theta(|\omega| - k_{ET}) \int \mathrm{d}\vec{r}_1 \int \mathrm{d}\vec{r}_2 \, \vec{E}_1(\vec{r}_1) \cdot \chi(\omega, \vec{r}_1, \vec{r}_2) \cdot \Delta\vec{E}_0(\vec{r}_2) \\ &= \int \mathrm{d}\omega \int \mathrm{d}\vec{r}_1 \int \mathrm{d}\vec{r}_2 \, \vec{E}_1(\vec{r}_1) \cdot \chi(\omega, \vec{r}_1, \vec{r}_2) \cdot \Delta\vec{E}_0(\vec{r}_2),\end{aligned} \tag{9}$$

which is a constant. That means, regardless of the value of k_{ET}, the following identity always holds

$$\Delta G_{sol}(k_{ET}) + \lambda_o(k_{ET}) = \Delta G^{eq}_{sol} + \lambda^{eq}_o, \tag{10}$$

where $\Delta G^{eq}_{sol} = \lim_{k_{ET}\to 0} \Delta G_{sol}(k_{ET})$ and $\lambda^{eq}_o = \lim_{k_{ET}\to 0} \lambda_o(k_{ET})$. This proves Equation 18 in the main text.

Supplementary Note 2: The generalized Fokker-Planck equation

In the literature, the solvent coordinate x, being the polarization energy, was often modeled as a random process, which followed the Brownian motion[3,4]. In the

work of Sumi and Marcus[5], the environmental motion, assumed to follow the Debye relaxation, was governed by the Fokker-Planck equation. However, for a general form of relaxations $S(t)$, a generalized approach needs to be taken.

We start with an overdamped generalized Langevin equation with a memory kernel $K(t)$[6],

$$\frac{\mathrm{d}x}{\mathrm{d}t} = -\int_0^t K(t-\tau)x(\tau)\mathrm{d}\tau + \xi(t), \tag{11}$$

where $\xi(t)$ is a colored noise with mean 0, $\langle \xi(t) \rangle = 0$. According to the fluctuation-dissipation theorem[7], the memory kernel $K(t)$ satisfies a relation with the noise $\xi(t)$,

$$K(\tau) = C\langle \xi(t+\tau)\xi(t) \rangle, \tag{12}$$

where the condition of stationarity is assumed. By Laplace transforming the Langevin equation of $x(t)$, we get

$$\hat{x}(z) = \frac{x(0)}{z+\hat{K}(z)} + \frac{\hat{\xi}(z)}{z+\hat{K}(z)}, \tag{13}$$

where $x(0)$ is the value of $x(t)$ at $t=0$, and

$$\hat{f}(z) = \int_0^\infty \mathrm{d}t\, f(t)e^{-zt}, \tag{14}$$

for a general function $f(t)$. By the inverse Laplace transform of Equation 13, we get

$$x(t) = x(0)S(t) + \int_0^t \mathrm{d}\tau\, S(t-\tau)\xi(\tau), \tag{15}$$

where $S(t)$ is the normalized auto-correlation function (TCF) of $x(t)$ (defined in Equation 14 in the main text), whose Laplace transform satisfies

$$\hat{S}(z) = \frac{1}{z+\hat{K}(z)}. \tag{16}$$

If $\xi(t)$ is a Gaussian process, $x(t)$ is also a Gaussian process with its mean $\bar{x}(t)$ and variance $A(t)$ given by

$$\bar{x}(t) = x(0)S(t),$$
$$A(t) = \langle (x(t) - x(0)S(t))^2 \rangle = C^{-1} \int_0^t \int_0^t \mathrm{d}t_1 \mathrm{d}t_2 \, S(t - t_1)S(t - t_2)K(t_1 - t_2). \quad (17)$$

The probability distribution function (pdf) $P(x, t|x(0), t = 0)$ is given by

$$P(x, t|x(0), t = 0) = (\frac{1}{2\pi A(t)})^{1/2} \exp\left(-\frac{(x - \bar{x}(t))^2}{2A(t)} \right). \quad (18)$$

It is straightforward to show that $P(x, t|x(0), t = 0)$ satisfies the differential equation[8],

$$\frac{\partial P}{\partial t} = \left(\frac{\dot{A}(t)}{2} - \frac{\dot{S}(t)}{S(t)} A(t) \right) \frac{\partial^2 P}{\partial x^2} - \frac{\dot{S}(t)}{S(t)} \frac{\partial}{\partial x}(xP), \quad (19)$$

In this expression, $\dot{A}(t)$ can be computed by

$$\begin{aligned} \dot{A}(t) \equiv \frac{\mathrm{d}A}{\mathrm{d}t} &= \frac{2}{C} S(t) \mathcal{L}^{-1}\{\hat{S}(z)\hat{K}(z)\} \\ &= \frac{2}{C} S(t) \mathcal{L}^{-1}\{\hat{S}(z)(\frac{1}{\hat{S}(z)} - z)\} \\ &= -\frac{2}{C} S(t)\dot{S}(t), \quad S(0) = 1, \end{aligned} \quad (20)$$

where $\mathcal{L}^{-1}\{\hat{f}(z)\}$ means the inverse Laplace transform of the function $\hat{f}(z)$. As a result,

$$A(t) = \frac{1}{C}(1 - S^2(t)), \quad (21)$$

where the constant C is given by

$$C = \lim_{t \to \infty} \frac{1}{A(t)} = \lim_{t \to \infty} \langle x^2(t) \rangle^{-1}. \quad (22)$$

Hence, the pdf of $x(t)$ follows the generalized Fokker-Planck equation,

$$\frac{\partial P}{\partial t} = D(t) \left(C^{-1} \frac{\partial^2 P}{\partial x^2} + \frac{\partial}{\partial x}(xP) \right), \quad (23)$$

where $D(t)$ is the time-dependent diffusion coefficient[9],

$$D(t) = -\frac{1}{S(t)}\frac{\mathrm{d}S(t)}{\mathrm{d}t}. \tag{24}$$

In the limit of $t \to \infty$, $A(t) \to C^{-1}$, the pdf of $x(t)$ becomes

$$P(x,t|x(0),t=0) \to P_{eq}(x) = (\frac{C}{2\pi})^{1/2}\exp\left(-\frac{C}{2}x^2\right). \tag{25}$$

Therefore, a statistical free energy function $F(x)$ can be defined through $P_{eq}(x)$ as follows,

$$P_{eq}(x) \propto \exp(-\frac{F(x)}{k_BT}), \tag{26}$$

which gives the form of $F(x)$ as

$$F(x) = \frac{k_BTC}{2}x^2. \tag{27}$$

Comparing this form with the free energy function defined in Equation $10a$ in the main text, we get

$$C^{-1} = 2k_BT\lambda_o(k_{ET}). \tag{28}$$

Finally, the generalized Fokker-Planck equation, which governs the local motions around the donor and acceptor, is given by

$$\frac{\partial P}{\partial t} = D(t)\left(2\lambda_o^\gamma k_BT\frac{\partial^2 P}{\partial x^2} + \frac{\partial}{\partial x}(xP)\right), \tag{29}$$

where $\lambda_o^\gamma \equiv \lambda_o(k_{ET})$. This proves Equation 24 in the main text.

Supplementary Note 3: Photo-excited ET reactions

In a photo-excited ET reaction, at $t = 0$, the local environment is in equilibrium with the ground state of the donor-acceptor pair with the electric field, $\vec{E}_0$. Hence we define the "frozen" polarization vector to be

$$\vec{P}_f(\vec{r}) = \int \mathrm{d}\omega \int \mathrm{d}\vec{r}'\,\chi(\omega,\vec{r},\vec{r}')\cdot\vec{E}_0(\vec{r}'), \tag{30}$$

and the origin of the solvent coordinate x_0 is shifted accordingly,

$$x_0 = -\int d\vec{r}\,\vec{P}_f(\vec{r}) \cdot \Delta\vec{E}_0(\vec{r}), \quad \Delta\vec{E}_0 = \vec{E}_1 - \vec{E}_2, \tag{31}$$

Then the solvent coordinate is shifted according to Equation 5. $\vec{E}_0$ differs slightly from the electric field of the excited state, $\vec{E}_1$. The difference can be approximately measured through the solvation experiment [10], which gives the stabilization energy, ΔE_{sol} (see Equation 15 in the main text). It is easy to see that the difference of the minima of the excited state and the ground state of the donor-acceptor pair, $\Delta x(k_{ET})$, has an approximate relation with ΔE_{sol} [11],

$$\begin{aligned}\Delta x(k_{ET}) &= \int d\omega\theta(|\omega| - k_{ET}) \int d\vec{r}_1 \int d\vec{r}_2\,(\vec{E}_1(\vec{r}_1) - \vec{E}_0(\vec{r}_1)) \cdot \chi(\omega, \vec{r}_1, \vec{r}_2) \cdot \Delta\vec{E}_0(\vec{r}_2) \\ &= \gamma \lim_{k_{ET}\to 0} \Delta x(k_{ET}) \approx \gamma\Delta E_{sol},\end{aligned} \tag{32}$$

where γ is given by (see Equation 16 in the main text)

$$\gamma = \int_{-\infty}^{+\infty} d\omega\, S(\omega)\theta(|\omega| - k_{ET}), \tag{33}$$

and $S(\omega)$ is the Fourier transform of $S(t)$. Similarly, the identity between $\Delta G_{sol}(k_{ET})$ and $\lambda_o(k_{ET})$ (see Equation 9) is modified because of the shift of $\vec{P}_f$,

$$\Delta G_{sol}(k_{ET}) + \lambda_o(k_{ET}) + \Delta x(k_{ET}) = \Delta G_{sol}^{eq} + \lambda_o^{eq} + \Delta E_{sol}. \tag{34}$$

This is Equation 28 in the main text.

The initial distribution of the local environment is in equilibrium with the electric field, $\vec{E}_0$. Equivalently, $P(x, t = 0)$ is centered at $x = 0$, by definition. According to the concept of restricted ensembles, the variance of $P(x, t = 0)$ is given by $2\lambda_o^{\gamma} k_B T$ because relaxation modes of the local environment, which are slower than the ET reaction, are inactive during the reaction time. Hence, the initial distribution $P(x, t = 0)$ is given by

$$P(x, t = 0) = \sqrt{\frac{1}{4\pi\lambda_o^{\gamma} k_B T}} \exp\left(-\frac{x^2}{4\lambda_o^{\gamma} k_B T}\right), \tag{35}$$

which is Equation 30 in the main text.

Supplementary Note 4: The correlation function with multiple exponential components

If the solvation correlation function $S(t)$ can be written as a sum of multiple exponential functions,

$$S(t) = \sum_{i=1}^{N} c_i e^{-t/\tau_i}, \tag{36}$$

where N is the number of components, the Laplace transform of $S(t)$, $S(\omega)$, reads (see Equation 13 in the main text),

$$S(\omega) = \frac{1}{\pi}\int_0^{+\infty} \mathrm{d}t\, S(t)\cos\omega t = \frac{1}{\pi}\sum_{i=1}^{N} c_i \frac{\tau_i}{1+(\omega\tau_i)^2}. \tag{37}$$

With a given value of k_{ET}, the dynamic factor γ can be computed using Equation 33,

$$\gamma = \int_{-\infty}^{+\infty} \mathrm{d}\omega\, S(\omega)\theta(|\omega| - k_{ET}) = 1 - \frac{2}{\pi}\sum_{i}^{N} c_i \arctan(k_{ET}\tau_i), \tag{38}$$

which is the same as Equation 31 in the main text.

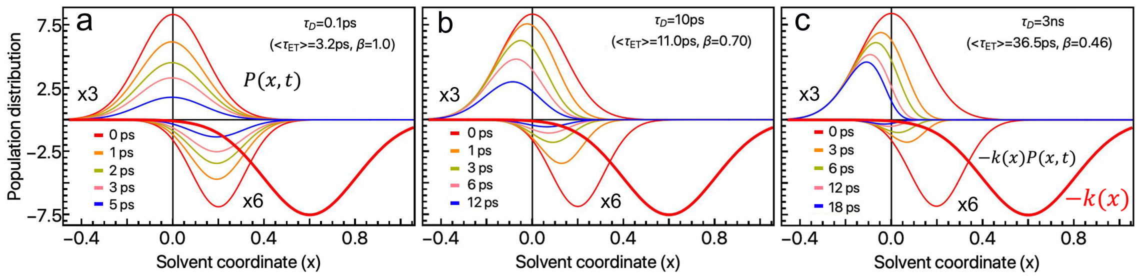

Supplementary Figure 1: The time evolution of reactants' distribution, $P(x,t)$, with different solvation timescales τ_D using the Sumi-Marcus model. Other values of parameters used are, $J = 0.020$eV, $\Delta G^o = -0.60$eV, $\lambda_i = 0.80$eV, and $\lambda_o = 0.40$eV, which are the same as Figure 2. The bolded red line represents the curve of $-k(x)$ without scaling. Within each graph, the upper panel displays the time evolution of $P(x,t)$, while the lower panel displays the evolution of the reaction rate's distribution, $-k(x)P(x,t)$. (a) $\tau_D = 0.1$ps. (b) $\tau_D = 10$ps. (c) $\tau_D = 3$ns.

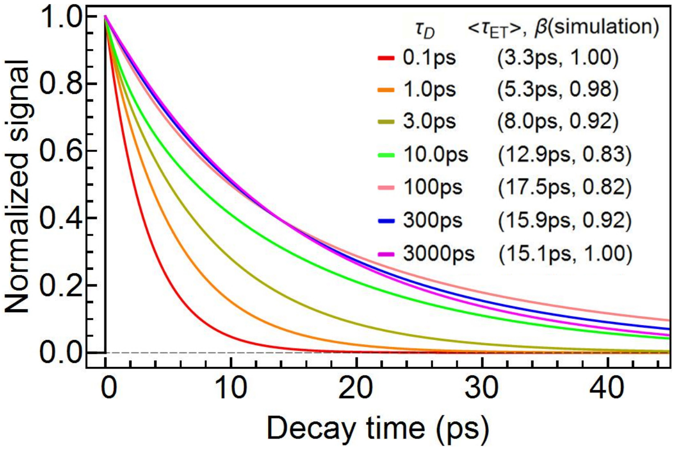

Supplementary Figure 2: Simulations of photo-excited ET dynamics with different solvation timescales, τ_D using the nonergodic model with the stablization energy, $\Delta E_{sol} = 0.025$eV. Other values used are the same as those in Supplementary Figure 1.

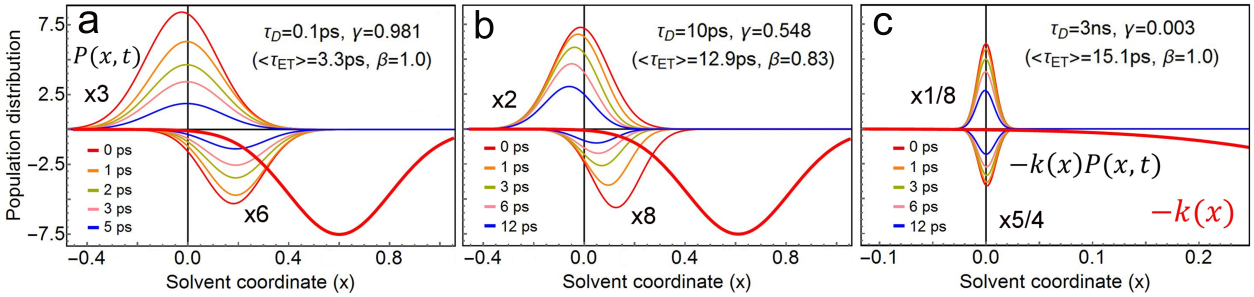

Supplementary Figure 3: The time evolution of reactants' distribution, $P(x,t)$, with different solvation timescales τ_D using the nonergodic model with the stablization energy, $\Delta E_{sol} = 0.025$eV. Values of parameters used are the same as those in Supplementary Figure 1. The bolded red line represents the curve of $-k(x)$ without scaling. Within each graph, the upper panel displays the time evolution of $P(x,t)$, while the lower panel displays the evolution of the reaction rate's distribution, $-k(x)P(x,t)$. (a) $\tau_D = 0.1$ps. (b) $\tau_D = 10$ps. (c) $\tau_D = 3$ns.

Supplementary References

1. Matyushov, D. V. Nonergodic activated kinetics in polar media. *J. Chem. Phys.* **130** (2009).

2. Palmer, R. G. Broken ergodicity. *Adv. Phys.* **31**, 669–735 (1982).

3. Kramers, H. A. Brownian motion in a field of force and the diffusion model of chemical reactions. *Physica* **7** (1940).

4. Calef, D. F. & Wolynes, P. G. Classical solvent dynamics and electron transfer. II. Molecular aspects. *J. Chem. Phys.* **78**, 470–482 (1983).

5. Sumi, H. & Marcus, R. A. Dynamical effects in electron transfer reactions. *J. Chem. Phys.* **84**, 4894–4914 (1986).

6. Risken, H. *The Fokker-Planck Equation* (Springer-Verlag Berlin Heidelberg, 1989).

7. Kubo, R. The fluctuation-dissipation theorem. *Rep. Prog. Phys.* **29** (1966).

8. Adelman, S. A. Fokker-Planck equations for simple non-Markovian systems. *J. Chem. Phys.* **64**, 124–130 (1976).

9. Hynes, J. T. Outer-Sphere Electron-Transfer Reactions and Frequency-Dependent Friction. *J. Phys. Chem.* **90**, 3701–3706 (1986).

10. Chang, C.-W. *et al.* Ultrafast solvation dynamics at binding and active sites of photolyases. *Proc. Natl. Acad. Sci. USA* **107**, 2914–2919 (2010).

11. Maroncelli, M. & Fleming, G. R. Computer simulation of the dynamics of aqueous solvation. *J. Chem. Phys.* **89**, 1988 (1988).
