# Supplementary material for: Effects of nonequilibrium fluctuations on ultrafast short-range electron transfer dynamics
Source: Nat Commun. 2020 Jun 4;11:2822. doi: 10.1038/s41467-020-15535-y (PMC7272615; doi:10.1038/s41467-020-15535-y)
Supplement: Supplementary file 2 — Peer Review [file 41467_2020_15535_MOESM2_ESM.pdf]

Reviewers' comments:

Reviewer #1 (Remarks to the Author):

This is an interesting application of the concept of nonergodic kinetics, which I believe deserves publication. The authors considered kinetics of ultrafast electron transfer between tryptophan and tyrosine residues and the photoexcited flavin cofactor. The observations suggest that the population dynamics is stretched-exponential for slower reactions with the semi-quinone form of flavin, but becomes single-exponential for faster reactions when flavin is prepared in the oxidized state. In order to explain these observations, the authors use the model in which the medium reorganization depends on the observation window. They, therefore, split the medium in a fast subsystem, which they identify with intramolecular vibrations (see below), and a slower subsystem, which can relax with the time-scales much slower than the reaction. The authors are able to quantitatively describe the observed kinetics and find some interesting and novel phenomenology regarding the stretched population dynamics. The paper is well written and deserves publication. I have a few questions that the authors might consider when revising the manuscript.

An interesting observations is the non-monotonic dependence of the stretching exponent on the medium relaxation time. The kinetics is exponential for long relaxation times, stretched for intermediate times, and returns back to exponential for fast medium relaxation. Since the relaxation time is a strong (Arrhenius) function of temperature, there is a possibility that such a nonlinear dependence of the stretching exponent might be observed when temperature is varied. I realize that this question has not been addressed in this set of experiments, but it might be a useful target for future measurements. The authors might comment on this opportunity.

It is probably important to realize the distinction between the restricted and dynamically restricted ensembles. Restricted ensembles were most clearly specified in the work of Palmer (Ref 22). They limit the phase space of the system by some well-defined constraints, such as symmetry requirements at phase transition of the bulk material. In contrast, the dynamically restricted ensemble introduced by Matyushov does not involve restrictions imposed by the internal symmetry of the problem, but utilizes the rate constant as the means to constrain the phase space. Therefore, in contrast to Palmer's formulation, the constraint is flexible and is moved as the rate of the reaction changes. It probably makes sense to stress that the authors use the "dynamically restricted" ensemble applicable to ultrafast nonergodic kinetics, in contrast to Palmer's restricted ensemble typically employed in glass science.

It is also important to better understand the initial conditions for the dynamic equations adopted by the authors (eqs (30) and (S35)). They adopted the nonergodic reorganization energy in these equations, which assumes restricted sampling. However, the ground state of flavin stays for very long time in equilibrium with the bath and one would anticipate that the entire reorganization energy, consistent with complete sampling of the phase space, should enter the initial conditions. Photoexcitation lifts this initial equilibrium distribution to the excited state, as was originally done in Sumi-Marcus theory, but the initial distribution should be characterized by equilibrium parameters.

The formulation of the Sumi-Marcus dynamics in eq (30) and the results of calculations listed in Table 1 assign the fast component of the medium reorganization to intramolecular vibrations. This does not have to be the case, and in fact the formalism is not sensitive to this assignment. This component likely represents the combined effect of all ballistic motions of the medium, which include both classical localized vibrations of the protein and inertial ballistic motions of water. According to measurements of Stokes-shift dynamics by Fleming and co-workers, ballistic relaxation of water is the main component of the Stokes-shift dynamics, at least for optical dyes dissolved in water. This change of physics might explain fairly large values of "internal reorganization energy" in Table 1. Those are not intramolecular vibrations but, instead, combined contributions of all fast nuclear modes.

Minor issues:

1. There is a typo in eq (3): the Franck-Condon factor in eq (2) needs to be normalized and the factor 1/(2 (pi lambda k T)^{1/2}) is missing from the equation. This omission does not affect authors' calculations; for instance, eq (23) is correct.

Reviewer #2 (Remarks to the Author):

The paper presents a theoretical model to describe the dynamics of ultrafast electron transfer (ET) reactions in complex biological environments. The proposed model is a combination of the well known Sumi-Marcus model, which takes into account dynamical effects of an additional ”diffusional” coordinate in ET, and the model of the dynamical arrest in ET developed by Matyushov, which essentially introduces the ET time scale dependent cutoff for the environmental spectral density. The main feature of the proposed model is the introduction of the dynamical factor γ characterizing the relationship between the rate of electron transfer and relaxation timescale of the environment. Ultimately the authors present the dynamical Smoluchowski-type equations for the reactant state population (probability density) with the reaction parameters depending on γ. Specifically, the reaction sink intensity is represented as a rate of a nonadiabatic transition in curve crossing formulation with the diabatic (crossing) free energy curves depending on the ET timescale itself and thus on the dynamical factor γ. As a result of γ-dependent reactant and product free energy surfaces, the reaction free energy and reorganization energy become γ-dependent as well, and that leads to nontrivial dynamical behavior depending on the value of γ. The authors also assume that the ET timescale can be considered as a parameter which makes the dynamical factor γ a parameter as well although in the original Matyushov’s model these two quantities are to be determined self consistently. Based on the values of γ the ultrafast ET processes are classified into three classes corresponding to frozen (fast ET), active (moderate ET), and equilibrium (slow ET) environments. The model is then used to demonstrate that in certain cases the dynamics might exhibit a behavior significantly different from a single exponential decay and described by a stretched exponential or multi-exponential laws.

In overall, the idea of incorporating the dynamical arrest model into Sumi-Marcus model for ET is quite interesting and certainly deserves attention. However, the way this idea is communicated in the submitted manuscript makes it impossible to recommend the paper for publication in Nature Communications. Listed below are the reasons for such a recommendation:

(1) The material in the paper is not really novel because both of the models (by Sumi-Marcus and by Matyushov) are well known and were discussed in the literature extensively.

(2) The presentation is too technical for an average reader of Nature Communications and in parts resembles excerpts from a textbook with well known relations. The technical parts of the paper are sometimes difficult to follow and the supplementary information does not provide enough details. That will make it impossible to reproduce the presented results or use the method for other calculations.

(3) It is not clear from the text how the authors obtained solutions of the dynamical equations for the reactant probability density (survival probability of the reactant state). I assume it has been done by numerically solving the differential equation but it would be very useful to provide at least a general description of the procedure.

(4) The connection to the experiments is very vague. The input parameters for the simulations were determined from experimental data but direct comparison to experimental kinetic measurements is provided. The relevant ”Applications” section is very short (less than a page long) and contains a very brief and general discussion of the experimental system.

(5) The text of the manuscript contains some minor grammatical and stylistic mistakes which have to be fixed.

In conclusion I would like to add that as mentioned before the model itself certainly deserves attention and if properly rearranged and rewritten the paper could be suitable for publication in another, more specialized journal.

[PDF file of this review attached]

Reviewer #3 (Remarks to the Author):

The manuscript develops a model of the kinetic behavior of short-range electron-transfer (ET) reactions in complex environments. The aim of the work is to provide a unified treatment of diverse regimes of coupling between a fluctuating environment and an ET process. Its main novelty is in the ability to address a variety of characteristic time scales, addressing both equilibrium conditions (for which classic Marcus theory provides an appropriate description) and non-equilibrium ones, which are shown to exhibit non-ergodic ET dynamics caused by the freezing of relaxation modes that are slower than the ET characteristic time scale. The coupling is described in the model by a dynamic factor gamma ranging from 0 (no coupling, with the ET process occurring in a frozen environment) and 1 (complete coupling between ET reaction and environment dynamics). The model indicates that, in general, the coupling causes a decrease of both the reaction free energy and the reorganization energy. As an application, two types of ET reactions occurring in flavodoxin are briefly addressed, in which experimental data on the ET dynamics is used to estimate the reorganization energies required to compute the time evolution of the reactant population [Eqn (29)].

This works makes an important contribution to the modeling and interpretation of photo-induced ET reactions in biology and it is very likely to have an immediate major impact in this field. In view of its generality, the model proposed could also be of great relevance in the study of a variety of electron transfer processes in chemistry, materials science and solution chemistry. It is a high quality paper addressing a complex and topical field of research of both fundamental and applied interest. In my opinion, the manuscript is suitable for publication in Nature Communication, but I list below a series of concerns that the authors may wish to address.

- One of the most obvious limitations of the model proposed stems from the rather coarse description of environmental fluctuations in terms of a purely diffusive process, as the authors mention themselves in the Conclusions. To what extent is this assumption affecting the model, and do the authors envisage potential ways to devise a more realistic description of complex fluctuating environments? It would be very helpful to have at least a short description of how the model could be improved from this perspective.

- The validation of the model is restricted to a single case (flavodoxin). It would be very helpful if the authors could present more examples of the applicability of the model to other photo-induced processes in proteins (and, ideally, other environments).

- From the flavodoxin example provided, it looks like the model proposed provides considerable insight into the ET reaction mechanism. It remains however unclear whether the model can provide a predictive tool, and, in particular, whether a fully ab initio approach to ET kinetics can be developed based , with no need for experimental data. Can the authors comment on the robustness of their method and, in particular, on whether their approach could be extended beyond a purely phenomenological description of the ET reaction dynamics.

Reviewer #4 (Remarks to the Author):

A theory of non-equilibrium electron transfer is highly interesting but challenging. The manuscript by Lu et al. reports their efforts on formulating such an effective non-equilibrium electron transfer theory. The reported formulation is largely based on previous works by Marcus&Sumi and Matyushov. To me the key difference compared to the earlier formulation is to rewrite the ET kinetics in terms of the so-called \gamma and nonergodic free energy parameters. These parameters quantify the impact of nonergodicity, and have been defined in the work of Matyushov (JCP, 2009). In terms of the ET kinetics, the major difference between the current study and previous work occurs in the so-called frozen regime, where the time-dependent reactant survival probability showed a single exponential behavior. This reactant decay kinetics is different from the multi-exponential decay predicted by Marcus and Sumi. The authors further applied their theory to investigate ultrafast ET in flavodoxin. While the research is interesting and the paper is well presented, I am not convinced the developed theory is much new and general. Compared to earlier developments, limited new physical insight can be inferred. Additionally, the theory developments targeted certain special conditions that limits the use of the theory in general non-equilibrium electron transfer processes. Therefore, the current manuscript is not suitable for publication in nature communication. Publication in a specialized journal is appropriate.

1. I am not sure that the single exponential decay in the frozen regime is general. The theoretical formulation is largely the same as prior work, but using the nonergodic free energy and reorganization energy defined based on the restricted ensembles. My intuition is that the single exponential decay holds exact only in the limit of 1/k_{ET}=0. (Please prove if this is not true) This can also be inferred from the previous formulation with the restricted ensembles (JCP, 4894, 1986). So, before reaching the 1/k_{ET}=0 limit, the decay kinetics is in principle multi-exponential, but the apparent decay behavior depends on the differences among those exponentials. In the numerical simulations of the manuscript, it showed that x=0 dominates (at the end of Page 17), but the none zero width of P(x,t) suggests the contributions from other x-dependent rate k(x). In the studied case, I believe that the x=0 term dominates, however, I am not sure this is still the case when relaxing those many strict approximations and considering the missing factors in deriving the analytical theory. There are only a small number of experiments that supported the single exponential decay. The physical explanation for the single exponential decay in the frozen regime is the direct consequence of the restricted ensembles and could be considered new. So, I trust the results presented in this study, but I don't think they are general. There are a few general theoretical formulations for the non-equilibrium kinetics (e.g. JCP, 103, 595 and chem. Sci. 5, 3761). Comparisons with those results may be useful.

2. The overall nonergodic impact to kinetics is to reduce the effective reaction free energy barrier (J. Mol. Liq. 266, 361). The further assignment of the non-equilibrium reaction free energy (in principle not well defined) to contributions from the reorganization energy and others components is somewhat empirical and depends on the detailed knowledge of dynamics of the system (e.g. solvent dynamics S(t) or S(\omega) to define \gamma in the current formulation). These could introduce some arbitrariness in the application. For example, in Table I, \lambda_o^{eq} varies from 0.14 eV to 0.33 eV for FMN for different mutants. I am not sure this is valid. \beta from fitting the experiments is valid, and overall effective free energy barrier inferred from experiments is useful. The further decomposition may not be unique.

3. Is the pre-factor given on page 3 for eq.(2) correct?

4. On page 4, the authors stated that "Due to large driving forces, the ET reaction ... happens within a few hundred femtoseconds ...". I think that ultrafast electron transfer of the cited ET reaction is caused by the near zero reaction free energy (or barrierless reaction), (\DeltaG + \lambda \approx 0, see the data in Table I). Not just the large driving force itself. Also, the DA coupling for the reaction is ~20 meV, the estimated coherent ET time (h/V) is about 200 fs, which is on the similar time scale of the observed ET rate of 300-400 fs. If coherence plays a sizable role in the ET kinetics, will the conclusions for FMN still robust?

Responses to review reports

REVIEWER 1

This is an interesting application of the concept of nonergodic kinetics, which I believe deserves publication. The authors considered kinetics of ultrafast electron transfer between tryptophan and tyrosine residues and the photoexcited flavin cofactor. The observations suggest that the population dynamics is stretched-exponential for slower reactions with the semi-quinone form of flavin, but becomes single-exponential for faster reactions when flavin is prepared in the oxidized state. In order to explain these observations, the authors use the model in which the medium reorganization depends on the observation window. They, therefore, split the medium in a fast subsystem, which they identify with intramolecular vibrations (see below), and a slower subsystem, which can relax with the time-scales much slower than the reaction. The authors are able to quantitatively describe the observed kinetics and find some interesting and novel phenomenology regarding the stretched population dynamics. The paper is well written and deserves publication. I have a few questions that the authors might consider when revising the manuscript.

Respond: We are very happy that the reviewer 1 recognizes our work and fully appreciates our systematic studies. We are so glad that the reviewer recommended the publication in nature Commons and we fully appreciate the insightful comments.

An interesting observation is the non-monotonic dependence of the stretching exponent on the medium relaxation time. The kinetics is exponential for long relaxation times, stretched for intermediate times, and returns back to exponential for fast medium relaxation. Since the relaxation time is a strong (Arrhenius) function of temperature, there is a possibility that such a nonlinear dependence of the stretching exponent might be observed when temperature is varied. I realize that this question has not been addressed in this set of experiments, but it might be a useful target for future measurements. The authors might comment on this opportunity.

Respond: This is a very good suggestion. For this beautiful system, we already plan to do temperature studies soon. The temperature effect on ET rate is a complicated issue and also has been studied in other systems for a while. Our group work has shown that all the relaxation components on the ultrafast timescales have temperature-dependent relaxation rates, which generally follows the Arrhenius law (PNAS, 113, 30 (2016)). Furthermore, it has been suggested that the solvent reorganization energy, λo, as well as the reaction free energy, ΔG^o, can also be temperature-dependent (JACS, 138, 29 (2016)). As the temperature T increases, the part of λo contributed by translational motions in the local environment will decrease with a rate proportional to 1/T , while the variation of ΔG^o is dependent on both the environment and the detailed structure of the donor and acceptor. In general, we may expect λo and ΔG^o have weaker temperature dependence compared to the solvation timescales. Therefore, we may assume λo and ΔG^o roughly stay constant within a certain range of temperature. On the other hand,

there should be significant variation in the solvation timescales within the same range of temperature. Under these conditions, when the ET rate is close to the rate of the solvation component with the largest coefficient (for example, it is the solvation component with a time constant of a few ps in flavodoxin), we may be able to observe a non-monotonic variation of the stretched factor β with varying temperature. We definitely will explore this effect.

It is probably important to realize the distinction between the restricted and dynamically restricted ensembles. Restricted ensembles were most clearly specified in the work of Palmer (Ref 22). They limit the phase space of the system by some well-defined constraints, such as symmetry requirements at phase transition of the bulk material. In contrast, the dynamically restricted ensemble introduced by Matyushov does not involve restrictions imposed by the internal symmetry of the problem, but utilizes the rate constant as the means to constrain the phase space. Therefore, in contrast to Palmer's formulation, the constraint is flexible and is moved as the rate of the reaction changes. It probably makes sense to stress that the authors use the "dynamically restricted" ensemble applicable to ultrafast nonergodic kinetics, in contrast to Palmer's restricted ensemble typically employed in glass science.

Respond: Thanks for pointing out the differences between restricted ensembles and dynamically restricted ensembles. We should also stress that this concept of *weak ergodicity breaking* is used in this model. It is understood in the sense that the phase space of which stays nonergodic within a finite time window ($t_w = 1/k_{ET}$ in this case), instead of staying for effectively infinite times (Leuzzi L. et. al., *Thermodynamics of the Glassy State*, Taylor & Francis). This ET model works as a thermodynamic approximation for an innate kinetic phenomenon. The work towards a truly kinetic model is under progress.

It is also important to better understand the initial conditions for the dynamic equations adopted by the authors (eqs (30) and (S35)). They adopted the non-ergodic reorganization energy in these equations, which assumes restricted sampling. However, the ground state of flavin stays for very long time in equilibrium with the bath and one would anticipate that the entire reorganization energy, consistent with complete sampling of the phase space, should enter the initial conditions. Photoexcitation lifts this initial equilibrium distribution to the excited state, as was originally done in Sumi-Marcus theory, but the initial distribution should be characterized by equilibrium parameters.

Respond: We want to take this opportunity to elaborate on the reason behind our choice for the initial condition of $P(x, t)$ in eqs (30) and (S35). According to the concept of (dynamically) restricted ensembles, given a reaction time window ($t_w = 1/k_{ET}$), the phase space of the system (the environment) is separated into a group of isolated phase spaces. We denote any isolated phase space by σ_i, $i = 1, 2, \ldots, N$, and the group of phase spaces by $\Sigma = \{\sigma_1, \sigma_2, \ldots, \sigma_N\}$. The union of all elements in Σ is the complete phase space of the environment. The model (eq (29)) describes the time evolution of the reactants' population within each *isolated phasespace*.

Based on our initial assumptions for developing the model of short-range ET reactions, we reason that the behaviors of the system within each isolated phase space are approximately the same. Firstly, we assume that the electronic coupling J is a constant, which is independent of the configuration of the environment. Secondly, since the donor and acceptor interacts with the environment *linearly*, the nonergodic free energy functions, F_1^γ and F_2^γ (eqs (19)), in each isolated phase space, only differs by a shift of the minimum along the x coordinate. The shift along the x coordinate comes from the polarization energy induced by the *inactive* relaxation modes in the environment which is the same for the reactant and product states. The difference between the minima of F_1^γ and F_2^γ gives the same value of ΔG^γ. As a result, the reaction kernel is the same for each σi in Σ. Consequently, the time evolution of the reactants' distribution in each σi follows the same differential equation (eq (29)).

However, whether these assumptions hold for ET reactions in general are subject to question. For example, it is possible that the distance between the donor and acceptor can vary because of local fluctuations. In other cases, the nonergodic free energy functions or the nonergodic driving force can be dependent on specific restricted phase spaces. The model has to be modified accordingly when applied to these situations.

The formulation of the Sumi-Marcus dynamics in eq (30) and the results of calculations listed in Table 1 assign the fast component of the medium reorganization to intramolecular vibrations. This does not have to be the case, and in fact the formalism is not sensitive to this assignment. This component likely represents the combined effect of all ballistic motions of the medium, which include both classical localized vibrations of the protein and inertial ballistic motions of water. According to measurements of Stokes-shift dynamics by Fleming and co-workers, ballistic relaxation of water is the main component of the Stokes-shift dynamics, at least for optical dyes dissolved in water. This change of physics might explain fairly large values of "internal reorganization energy" in Table 1. Those are not intramolecular vibrations but, instead, combined contributions of tall fast nuclear modes.

Respond: It is true that the inner reorganization energy defined in this work (eq (30)), λ_i, can include contributions from all the fast nuclear modes inside the donor and acceptor as well as in the environment that is not resolved in the solvation correlation function $S(t)$. However, we think that the value of λ_i for each mutant, in Table 1, is mostly contributed by the intramolecular vibrational modes inside the donor and acceptor, for the following reasons.

a. The functional site of flavodoxin is buried inside the binding pocket with partial exposure to the protein's surface (JACS, 132, 12741(2010)). The measured solvation dynamics around the functional site, including effects of surrounding motions up to 7-10 Å, does not have sub-ps components, while typical ballistic motions in solvent are resolved in tens of fs to hundreds of fs timescale (see for example Chem. Phys. Lett., 388, 120 (2004)). We have systematically measured the water motions around the protein surface (for the recent work, see PNAS, 113, 8424 (2016)) and especially the motions of the first-layer hydration water occur at least in a few picoseconds. Hence, the distant ballistic motions of free water, relative to the donor and acceptor at

the functional site, do not have large effects on the ET dynamics.

b. On the other hand, it has been suggested that there is a significant structural change between the oxidized flavin, FMN, in which the flavin ring has a planar configuration, and the negatively charged, FMN^{-}, whose flavin ring is slightly bent. The flavin in the hydroquinone form, $FMNH^{-}$ or $FMNH_2$, is bent by a larger angle compared to the semiquinone. This structural change can result in large contributions to the inner reorganization energy (JACS, 118, 9402(1996); JACS, 130, 7695(2008)).

There is a typo in eq (3): the Franck-Condon factor in eq (2) needs to be normalized and the factor $(\pi\lambda k_B T)^{-1/2}$ is missing from the equation. This omission does not affect authors' calculations; for instance, eq (23) is correct.

Respond: Thanks for pointing it out. Instead, eq (2) should be

$$k_{ET} = \frac{1}{\tau_{ET}} = A\sqrt{\frac{1}{4\pi k_B T\lambda}}e^{-\frac{(\Delta G^o+\lambda)^2}{4k_B T\lambda}}$$

REVIEWER 2

The paper presents a theoretical model to describe the dynamics of ultrafast electron transfer (ET) reactions in complex biological environments. The proposed model is a combination of the well-known Sumi-Marcus model, which takes into account dynamical effects of an additional "diffusive" coordinate in ET, and the model of the dynamical arrest in ET developed by Matyushov, which essentially introduces the ET time scale dependent cutoff for the environmental spectral density. The main feature of the proposed model is the introduction of the dynamical factor γ characterizing the relationship between the rate of electron transfer and relaxation timescale of the environment. Ultimately the authors present the dynamical Smoluchowski-type equations for the reactant state population (probability density) with the reaction parameters depending on γ. Specifically, the reaction sink intensity is represented as a rate of a nonadiabatic transition in curve crossing formulation with the diabatic (crossing) free energy curves depending on the ET timescale itself and thus on the dynamical factor γ. As a result of γ-dependent reactant and product free energy surfaces, the reaction free energy and reorganization energy become γ-dependent as well, and that leads to nontrivial dynamical behavior depending on the value of γ. The authors also assume that the ET timescale can be considered as a parameter which makes the dynamical factor γ a parameter as well although in the original Matyushov's model these two quantities are to be determined self consistently. Based on the values of γ the ultrafast ET processes are classified into three classes corresponding to frozen (fast ET), active (moderate ET), and equilibrium (slow ET) environments. The model is then used to demonstrate that in certain cases the dynamics might exhibit a behavior significantly different from a single exponential decay and described by a stretched exponential or multi-exponential laws. In overall, the idea of incorporating the dynamical arrest model into Sumi-Marcus model for ET is quite interesting and certainly deserves attention.

Respond: We greatly appreciate the reviewer 2 for the careful report and the efforts. Basically, the reviewer 2 fully understands the key points in the manuscript. Clearly, the reviewer 2 is an expert in (ET) theory and we are very happy to discuss deeply with the reviewer 2 to improve our manuscript.

However, the way this idea is communicated in the submitted manuscript makes it impossible to recommend the paper for publication in Nature Communications. Listed below are the reasons for such a recommendation:

The material in the paper is not really novel because both of the models (by Sumi-Marcus and by Matyushov) are well known and were discussed in the literature extensively.

Respond: We do not agree with the reviewer 2 and we did not simply put the two theories together as the reviewer 2 can tell. On the other side, we need to develop a straightforward model to explain our experimental observations. We are not a

theoretical group and we would like to have a good model that can be directly applied to explain our experimental data. From the analyses of our data with the developed model, we are able to extract deep molecular meaning of various ET dynamics in biological systems.

Our simulations showed that the Sumi-Marcus model predicts slower ET dynamics with a longer solvation timescale while other parameters being fixed (see Figure 2B in the modified manuscript). On the other hand, with an increasing value of τ_D, the stretched factor β in the decay function, $e^{-\left(\frac{t}{\tau_{ET}}\right)^{\beta}}$, is monotonically decreased, as a result of the static heterogeneity of the environment. *This is in contradiction to the ultrafast ET dynamics between the photoexcited FMN and the tryptophan (tyrosine) residual, which we observed in flavodoxin.* The ET dynamics displays a single-exponential decay when the local motions are much slower than the reaction. To understand this novel dynamics, we proposed the nonergodic model by integrating the Sumi-Marcus model with the idea originally proposed by Matyushov. Clearly, we are not *simply* using two models but we developed the extended model with a proposed dynamic parameter γ, which is very transparent and easily understood, especially the varying reorganization energy and reaction free energy. Our simulations with this model showed that the stretched factor β changes non-monotonically with increasing τ_D (Figure 2A). Particularly, the characteristics of the dynamics in the frozen region, as predicted by the nonergodic model, is qualitatively different from what is obtained from the Sumi-Marcus model (see Figure 2 and compare Figure 3 with Figure S1).

We think that this model provides a novel and comprehensive description over ET dynamics on the ultrafast timescales (the active and frozen regions), which successfully explains our experimental results, while being also consistent with the Marcus theory for ET dynamics in the equilibrium region. Clearly, these new developments and applications are significant to many ET reactions in biology and will have wide applications in materials, chemistry and biology.

The presentation is too technical for an average reader of Nature Communications and in parts resembles excerpts from a textbook with well-known relations. The technical parts of the paper are sometimes difficult to follow and the supplementary information does not provide enough details. That will make it impossible to reproduce the presented results or use the method for other calculations.

It is not clear from the text how the authors obtained solutions of the dynamical equations for the reactant probability density (survival probability of the reactant state). I assume it has been done by numerically solving the differential equation but it would be very useful to provide at least a general description of the procedure.

Respond: The following part is also added to the SI. In this work, we used the software called *Mathematica* to solve the differential equations. The software provides numerical methods for solving ordinary or partial differential equations, such as NDSolve, which are simple to use. The solution of NDSolve is then numerically integrated using methods, such as NIntegrate, to give $Q(t)$ (eq (4)). $Q(t)$ can be fitted by a stretched-exponential function using the method, NonlinearModelFit.

The connection to the experiments is very vague. The input parameters for the simulations were determined from experimental data but direct comparison to experimental kinetic measurements is provided. The relevant "Applications" section is very short (less than a page long) and contains a very brief and general discussion of the experimental system

Respond: Thanks for the suggestions. In these simulations, most of the input parameters are obtained from other sources independent of the experimental data of ET reactions. For example, the reaction free energy ΔG^o for each mutant is obtained from electrochemical experiments (Biochemistry, 33, 8505, (1994); JPC, 95, 3416, (1991)). The solvation correlation function $S(t)$ is obtained from the solvation experiments (JACS, 132, 12741, (2010)). The parameters to be determined by our simulations are λ_i, which is assumed to be invariant under mutation, and λ_o^γ for each mutant.

Then we computed the driving force ΔG^γ using simulated results and compared it with the equilibrium value ΔG^o. We also compared λ_o^γ in the ET reaction involving FMN with λ_o^γ of $FMNH^\bullet$. Based on our reasoning, these comparisons show that in nonequilibrium ET reactions (active and frozen regions) the reaction driving force and the outer reorganization energy can significantly deviate from their equilibrium values, suggesting incomplete solvation of the local environment, which supports our final conclusions.

As for the "Applications" section, part of the experimental results is introduced in the "Introduction" section. For your convenience, we attach the modified "Applications" section in this response.

In this section, the model (Equation (29)) is applied to analyzing two types of photo-excited ET reactions in flavodoxin. As discussed, to get an accurate picture of the ET reaction, detailed information of the solvation dynamics is required. The ET reactions to be analyzed are between a photo-excited flavin cofactor (FMN^* or $FMNH^{\bullet *}$) and a nearby tryptophan residue in flavodoxin. The TCF of their solvation dynamics is in the form of

$$S(t) = c_1 e^{-t/\tau_1} + c_2 e^{-t/\tau_2} + c_3 e^{-t/\tau_3}$$

with τ_1 being a few ps, τ_2 being tens of ps, and τ_3 being hundreds of ps. The solvation dynamics does not have sub-picosecond components, which are often attributed to ballistic motions of bulk water, because the functional site in which the solvation is measured in these studies is buried deeply inside the binding pocket and is distant from the bulk water. The experimental solvation dynamics of flavodoxin with $FMNH^\bullet$ does not have a third component due to the shorter lifetime of $FMNH^\bullet$. Within each system, mutants are chosen such that each mutant is mutated at the same site and does not drastically change the protein's conformation and the structures of the donor and acceptor. It is therefore assumed that the solvation TCF, $S(t)$, and the inner reorganization energy, λ_i, stay invariant under mutation. Given that the data of each mutant's "equilibrium" free energy, ΔG^o, is available, the reorganization energies, λ_o^γ and λ_i, can be obtained by fitting the experimental ET dynamics with Equation (29).

The ET reaction of FMN is in the frozen region and displays a single-exponential dynamics with the reaction rate being in the hundreds of fs, which is faster than those of local motions. As expected, the outer reorganization energy for each mutant is close to 0, a characteristic of ET reactions in the frozen region (Table 1 and Figure 5A). The large reaction rate is a result of low activation energy, being determined by the large driving force, ΔG^γ, as well as a fairly large inner reorganization energy, λ_i. It has been suggested that the unusual value of λ_i comes from the significant structural change between FMN and the negatively charged, FMN^-. On the other hand, the ET dynamics with $FMNH^\bullet$, being in the active region, is stretched with its reaction timescale comparable to the

shortest solvation timescale, τ_1. The model gives a small but nontrivial λ_o^γ (Table 1 and Figure 5B). From Table 1, it is obvious that the calculated reaction free energy ΔG^γ deviates significantly from its equilibrium value for each mutant.

In contrast to the predictions made by the Sumi-Marcus model (Figure 2B and Figure S1), the lack of strong dependencies on the static heterogeneity of the environment in the experimental results suggests that, for ultrafast short-range ET reactions, the electronic and vibrational structures of the donor and acceptor are insensitive to the fluctuations of the local environment. The influences of environmental fluctuations on the ET dynamics are reflected in their contributions to the outer reorganization energy as well as the driving force. Conversely, if the ET dynamics in the frozen region displays non-exponential decay, it might suggest that the donor and acceptor is strongly coupled with the environment such that the configurations of the surrounding molecules have an impact on the electronic and vibrational structures of donor and acceptor.

The text of the manuscript contains some minor grammatical and stylistic mistakes which have to be fixed. In conclusion I would like to add that as mentioned before the model itself certainly deserves attention and if properly arranged and rewritten the paper could be suitable for publication in another, more specialized journal.

Respond: Thanks again for the advice. We will work carefully to correct these mistakes.

We also want to add that our focus on this work was not to propose a new theory of ET reactions. Instead, we were trying to understand a new class of ET dynamics observed in the ultrafast regime from our experiments. There is no directly applicable model that can be transparently used by experimentalists. Our group has recently studied multiple short-range ET reactions in biological systems, most of which do not fit in the classic physical picture. This work improves our current understanding of ET dynamics in this new horizon, which is fundamental to our comprehension of molecular mechanisms behind many biological functions. Thus, based on the above arguments, we believe the work is a breakthrough in links between the experimental ultrafast ET in biology and the theoretical understanding of molecular mechanism using a newly developed extended model. We think the work is better suitable to be published in Nature Communications due to the large readers and our work absolutely can be applicable to many ET reactions in various fields.

REVIEWER 3

The manuscript develops a model of the kinetic behavior of short-range electron-transfer (ET) reactions in complex environments. The aim of the work is to provide a unified treatment of diverse regimes of coupling between a fluctuating environment and an ET process. Its main novelty is in the ability to address a variety of characteristic time scales, addressing both equilibrium conditions (for which classic Marcus theory provides an appropriate description) and non-equilibrium ones, which are shown to exhibit non-ergodic ET dynamics caused by the freezing of relaxation modes that are slower than the ET characteristic time scale. The coupling is described in the model by a dynamic factor gamma ranging from 0 (no coupling, with the ET process occurring in a frozen environment) and 1 (complete coupling between ET reaction and environment dynamics). The model indicates that, in general, the coupling causes a decrease of both the reaction free energy and the reorganization energy. As an application, two types of ET reactions occurring in flavodoxin are briefly addressed, in which experimental data on the ET dynamics is used to estimate the reorganization energies required to compute the time evolution of the reactant population [Eqn (29)].

Respond: We appreciate the reviewer 3 for the very positive report and fully appreciate the reviewer's efforts for reviewing our work.

This work makes an important contribution to the modeling and interpretation of photo-induced ET reactions in biology and it is very likely to have an immediate major impact in this field. In view of its generality, the model proposed could also be of great relevance in the study of a variety of electron transfer processes in chemistry, materials science and solution chemistry. It is a high-quality paper addressing a complex and topical field of research of both fundamental and applied interest. In my opinion, the manuscript is suitable for publication in Nature Communication, but I list below a series of concerns that the authors may wish to address.

Respond: Thank the reviewer 3 again for highly supporting our work suitable for publication in Nature Communication.

One of the most obvious limitations of the model proposed stems from the rather coarse description of environmental fluctuations in terms of a purely diffusive process, as the authors mention themselves in the Conclusions. To what extent is this assumption affecting the model, and do the authors envisage potential ways to devise a more realistic description of complex fluctuating environments? It would be very helpful to have at least a short description of how the model could be improved from this perspective.

Respond: The work in our group has shown that the local environment of a typical functional site has a rugged free energy surface with a hierarchy of energy barriers that result in multiple relaxation timescales (PNAS, 113, 30 (2016)). The energy barriers are in the order of a few $k_B T$, which can even be higher than the activation energy of ultrafast ET reactions. However, the current work on ET reactions still models the local fluctuations as diffusions over a smooth energy surface. Despite this drastic difference in describing local fluctuations, it is not clear how much difference a better model of

local fluctuations can make to the simulated ET dynamics.

Nevertheless, we are working towards an ET model which includes a more realistic description of environmental fluctuations. We expect that this new model can achieve the following results. Firstly, the model can account for the temperature dependence of various relaxation timescales. Secondly, this model can address nonergodic effects dynamically, which means it does not rely on an empirical parameter that is dependent on the ET reaction rate.

The validation of the model is restricted to a single case (flavodoxin). It would be very helpful if the authors could present more examples of the applicability of the model to other photo-induced processes in proteins (and, ideally, other environments).

Respond. The flavodoxin is an ideal model system for the validation of the model for several reasons.

a. The flavin inside the flavodoxin has two oxidation states (FMN and FMNH$^{\bullet}$), both of which can serve as the electron acceptor for a photoinduced ET reaction. The two reactions display different dynamics with different reaction rates, which are representatives of reactions in the frozen and active regions.

b. The local fluctuations for both reactions have been resolved experimentally. The local motions for the two ET reactions are very similar.

c. The reduction potential of each mutant used in this work (Table 1) has been measured experimentally.

With all these conditions available, we were able to make the important comparisons between ΔG^{γ} and ΔG^{o} for each mutant, and between the values of λ_o^{γ} for FMN and FMNH$^{\bullet}$. We were then able to draw the conclusions accordingly. However, in general, for a given system, it is difficult to obtain all the experimental data listed above. Lack of certain data could result in more free parameters that have to be determined by simulations, which would possibly weaken the validity of the model. Nonetheless, we have studied a series of ET reactions in different biological systems and we will apply the current model and improve its applicability in the near future work.

From the flavodoxin example provided, it looks like the model proposed provides considerable insight into the ET reaction mechanism. It remains however unclear whether the model can provide a predictive tool, and, in particular, whether a fully ab initio approach to ET kinetics can be developed based, with no need for experimental data. Can the authors comment on the robustness of their method and, in particular, on whether their approach could be extended beyond a purely phenomenological description of the ET reaction dynamics.

Respond: In principle, the spectral density $S(\omega)$ or the correlation function of the polarization energy $S(t)$ (eq (14)) can be computed using ab initio methods (JPCB, 116, 10294 (2012)). On the other hand, it is also possible to compute the electronic coupling (J), the driving force (ΔG^o) and reorganization energies (λ_i and λ_o) for a given system (JACS, 138, 1904, (2016)). These parameters can then be inserted into the model (eq (29)).

In this case, the value of γ (eq (16)) has to be obtained by iteratively solving the differential equation, since the average reaction time (τ_{ET}) is unknown. The self-consistent solution of the differential equation (eq (29)) will give the ET dynamics of the system. This approach of modeling ET dynamics can help us address important questions that cannot be easily answered by experimental methods. For example, photoinduced ET reactions involving the flavin molecule often have large λ_i. It is suggested that it is mostly contributed by the bending motion of the flavin ring (JACS, 130, 7695 (2008)), which is hard to prove experimentally.

REVIEWER 4

A theory of non-equilibrium electron transfer is highly interesting but challenging. The manuscript by Lu et al. reports their efforts on formulating such an effective non-equilibrium electron transfer theory. The reported formulation is largely based on previous works by Marcus&Sumi and Matyushov. To me the key difference compared to the earlier formulation is to rewrite the ET kinetics in terms of the so-called γ and nonergodic free energy parameters. These parameters quantify the impact of nonergodicity, and have been defined in the work of Matyushov (JCP, 2009). In terms of the ET kinetics, the major difference between the current study and previous work occurs in the so-called frozen regime, where the time-dependent reactant survival probability showed a single exponential behavior. This reactant decay kinetics is different from the multi-exponential decay predicted by Marcus and Sumi. The authors further applied their theory to investigate ultrafast ET in flavodoxin.

While the research is interesting and the paper is well presented, I am not convinced the developed theory is much new and general. Compared to earlier developments, limited new physical insight can be inferred. Additionally, the theory developments targeted certain special conditions that limits the use of the theory in general non-equilibrium electron transfer processes. Therefore, the current manuscript is not suitable for publication in nature communication. Publication in a specialized journal is appropriate.

Respond: Frist, I fully appreciate the reviewer's time and efforts on our work. I have to emphasize here that we are not a theory group and we did not aim to create a new theory in this work. What we did is that we like to develop a practical ET model based on previous theories/models to explain our observed experimental results. Currently, we did not find a theory or model in literature that can perfectly explain our data. Thus, we combined two powerful, previous models and developed an extended model to explain our data. Then, from our systematic studies, we can extract insightful knowledge about the molecular mechanism of ultrafast ET reactions. In turn, we can improve our new model again. Here, we showed the nice extended model can truly explain our observed ultrafast ET reactions. Thus, we do think the paper is better suitable to Nature Communication due to the significance of the extended model that can be used to explain a variety of ET reactions in material, chemistry and biology.

I am not sure that the single exponential decay in the frozen regime is general. The theoretical formulation is largely the same as prior work, but using the nonergodic free energy and reorganization energy defined based on the restricted ensembles. My intuition is that the single exponential decay holds exact only in the limit of $1/k_{ET} = 0$. (Please prove if this is not true) This can also be inferred from the previous formulation with the restricted ensembles (JCP, 4894, 1986). So, before reaching the $1/k_{ET} = 0$ limit, the decay kinetics is in principle multi-exponential, but the apparent decay behavior depends on the differences among those exponentials. In the numerical simulations of the manuscript, it showed that $x = 0$ dominates (at the end of Page 17), but the none zero width of $P(x, t)$ suggests the contributions from other x-dependent rate $k(x)$. In the

studied case, I believe that the $x = 0$ term dominates, however, I am not sure this is still the case when relaxing those many strict approximations and considering the missing factors in deriving the analytical theory. There are only a small number of experiments that supported the single exponential decay. The physical explanation for the single exponential decay in the frozen regime is the direct consequence of the restricted ensembles and could be considered new. So, I trust the results presented in this study, but I don't think they are general. There are a few general theoretical formulations for the non-equilibrium kinetics (e.g. JCP, 103, 595 and Chem. Sci. 5, 3761). Comparisons with those results may be useful.

Respond: The single-exponential decay in the frozen region is a result of the following assumptions:

a. Firstly, it is assumed that the electronic coupling between states J is a constant such that it does not vary with different configurations of the system.

b. Secondly, it is assumed that the configuration of the environment does not affect the sum of the driving force and the total reorganization energy, $\Delta G^o + \lambda$. This is a result of the assumption that the donor and acceptor interact with the environment through linear dipole-dipole interactions.

If either of these assumptions is broken, the ET dynamics in the frozen region will not be exponential. In the short-range ET reactions that we are concerned with in this work, these assumptions generally hold true. Moreover, by a few simple modifications, the nonergodic model can be applied to cases in which these assumptions are invalid.

This work helped us establish a different physical picture concerning the effects of local environment on the ET dynamics in the ultrafast regime. It has been understood that the free energy surface of a biological system (more generally, it can be any complex system) is rugged, with a hierarchy of energy barriers that result in multiple relaxation timescales (PNAS, 113, 30 (2016)). This is in contrast to the simple potential function of the reaction coordinate we usually assumed for an ET reaction, which puzzled us greatly. The nonergodic model helped us understand that the high energy barriers forbid samples that start at a certain part of the phase space from reaching other parts within a given time window ($t_w = 1/k_{ET}$). As a result, we can still use the reaction coordinate that has a simple potential function but with its curvature modified by the nonergodic effect.

We recognized the important contributions of related work (such as JCP, 103, 595, (1995) and Chem. Sci. 5, 3761, (2014)) to our understanding of nonequilibrium ET reactions. However, we think that the model in this work provides a novel and comprehensive description over ET dynamics on the ultrafast timescales, (the active and frozen regions). The description is also consistent with our experimental results about which no satisfactory explanations have been made by far in literature.

The overall nonergodic impact to kinetics is to reduce the effective reaction free energy barrier (J. Mol. Liq. 266, 361). The further assignment of the non-equilibrium reaction free energy (in principle not well defined) to contributions from the reorganization energy and other components is somewhat empirical and depends on the detailed knowledge of dynamics of the system (e.g. solvent dynamics $S(t)$ or $S(\omega)$ to define γ in the current formulation). These could introduce some arbitrariness in the

application. For example, in Table I, λ_o^γ varies from 0.14eV to 0.33eV for FMN for different mutants. I am not sure this is valid. β from fitting the experiments is valid, and overall effective free energy barrier inferred from experiments is useful. The further decomposition may not be unique.

Respond: The decomposition of the reorganization energy and the reaction free energy is based on the relative timescales of various local motions as compared to the reaction timescale. Therefore, it is possible that the inner reorganization energy λ_i can include contributions from fast environmental motions that are not resolved by the solvation experiment. However, in flavodoxin, the functional site of flavodoxin is buried inside the binding pocket with partial exposure to the protein's surface (JACS, 132, 12741(2010)). The measured solvation dynamics around the functional site, including effects of surrounding motions up to 10 Å, does not have sub-ps components, while typical ballistic motions are resolved in the ten of fs to hundreds of fs timescale (see for example Chem. Phys. Lett., 388, 120 (2004)). Hence, the fast, ballistic motions of water should not have important contributions to λ_i. We thus believe the decomposition of the reorganization energy is reasonable (also see reply to the reviewer 2 and see our hydration work).

On the other hand, we agree that the estimated value of λ_o^{eq} for each mutant for FMN is questionable, since in this case γ and λ_o^γ are both very small. Nevertheless, $\lambda_o^\gamma \approx 0$ does tell us the ET reaction is in the frozen region and the local environment barely moves during the reaction time, in comparison to the case of $FMNH^\bullet$. We think that even though the relevant parameters obtained by simulations, such as λ_i, λ_o^γ, and ΔG^γ, may not be completely quantitatively accurate, this kinetic approach of modeling ultrafast ET dynamics has its value, and can be used to analyze experimental results and obtain very insightful information. Also, we cannot solve all questions in one paper. More work is underway to test our recent various ET reactions in different biological systems using the newly developed model.

Is the pre-factor given on page 3 for eq.(2) correct?

Respond. We apologize for the mistake. Instead, it should be

$$k_{ET} = \frac{1}{\tau_{ET}} = A\sqrt{\frac{1}{4\pi k_B T\lambda}}\, e^{-\frac{(\Delta G^o+\lambda)^2}{4k_B T\lambda}}$$

On page 4, the authors stated that "Due to large driving forces, the ET reaction . . . happens within a few hundred femtoseconds ...". I think that ultrafast electron transfer of the cited ET reaction is caused by the near zero reaction free energy (or barrierless reaction), ($\Delta G + \lambda \approx 0$, see the data in Table I). Not just the large driving force itself. Also, the DA coupling for the reaction is ~ 20 meV, the estimated coherent ET time (h/V) is about 200 fs, which is on the similar time scale of the observed ET rate of 300 – 400fs. If coherence plays a sizable role in the ET kinetics, will the conclusions for FMN still robust?

Respond: Thanks for pointing it out. The sentence was not well phrased. We modified it

in the revised manuscript.

We did consider the potential roles of electronic and vibrational coherences, but we did not include these discussions into this paper. Firstly, “long-lived” electronic coherence often leads to oscillating population dynamics, but we did not observe any oscillating pattern in the data (Figure 5). Secondly, we argue that even if coherence is involved, the nonergodic effects on the ET dynamics still exist and the general conclusions about these effects should be qualitatively applicable to ET reactions in the frozen and active regions. Nonetheless, we are interested in examining the potential effects of coherence on ultrafast ET dynamics in the future work.

Reviewers' comments:

Reviewer #1 (Remarks to the Author):

After reading the authors' rebuttal and the revised manuscript, I believe my concerns have been mostly addressed/explained. The problem studied here is obviously both important and immensely complex. The authors represent a world's leading group in ultrafast dynamics of protein electron transfer and the issues still not fully resolved in this study reflect the state of the art in this field as is seen by the front runners. I think that the analysis presented in this paper is a robust trade-off between the desire for a quantitative description of the kinetic data and a limited set of established parameters for this complex system. It provides other practitioners with an example of a successful and practical theoretical tool which can be implemented for other kinetic studies.

After reading the comments by other reviewers, I respectfully disagree with the notion that the theory application presented in this paper does not provide new theoretical isights. It is true that the basic theoretical concepts used in this paper have been laid out in the past. However, application of the these general concepts to problems and systems as complex as protein electron transfer has been a very painful process. Even the Sumi-Marcus theory, which provided a very general framework for addressing competing time-scales back in 1986, has only recently started to find its applications in analyzing experiment. Clear-cut nonergodic effect in biology realistically have not been confirmed experimentally and, from this general standpoint of the state of the field, this paper presents a truly breakthrough development. There are potentially many more kinetic data falling into the realm of dynamical freezing and glassiness, which have not been appreciated as such, and still are waiting to be re-discovered because of the lack of direct and clear applications of abstract theoretical ideas to specific kinetic studies. I believe this paper paves the way to many such applications in the future. One has to realize that glassiness of proteins has been recognized starting from classical studies by Frauenfelder and Wolynes and has been in various ways entertained by a number of scholars. What does it mean practically in terms of specific rate measurements has been less clear. This paper offers exactly this perspective. I'm certain that many practitioners will find it more valuable than the abstract theoretical ideas entertained by theorists. The application of the theory to kinetic data also poses questions which future theories must answer and, in this way, also forces the theorists to refine their models (the most fruitful way the exchange of ideas should happen in science). The reviewers, in fact, raise some of these valuable suggestions pointing that this is not the end of the road and these results will help others to move forward.

p. 25: The authors insist that the free energy of electron transfer should show some ruggedness when calculated "correctly". While this notion has been advocated in glass science, one has to realize that it has been applied to the configuration landscape. This is the potential energy of the system as a function of all 3N coordinates, and it must be rugged as evidenced by computer experiments. However, in the case of electron transfer, all 3N coordinates of the system are projected on a single collective electron-transfer reaction coordinate. There is no evidence in the literature, including direct calculations of such free energy surfaces by molecular dynamics simulations, that this projection should preserve the ruggedness of the original U(r_3N) surface. The authors should either clearly indicate the evidence for such a claim or remove it altogether. The model used by the authors is already more complex than "simple diffusion" since the diffusion coefficient is time-dependent. Theoretical work has shown that this model is equivalent to Langevin dynamics with stretched-exponential memory kernel. Do we have any present evidence that more complex models are required? I think the first paragraph on p.25 should be changed. The current write-up suggests that the model used in this paper is not adequate for this problem, while there is no evidence that this is true. In fact, the fitting of the experimental results presented in the paper suggests that the model is sufficient to describe ultra-fast electron transfer in proteins.

Minor corrections:

1. p5, first par: remove break between sentences
2. eq 9: the authors use 1 and 2 for the initial and final states, as well as R and P. It would be useful if they made connection between the two conventions, such a 1=R, 2=P.
3. I suggest that the authors find ways to remind the reader the meaning of frozen/active/equilibrium through the manuscript. This is particularly true for those who are familiar with Marcus' normal/inverted classification. For instance, on p.23, "being in the active region" might follow with the reference like (Fig. 1B, II).
4. p. 23, 2nd line from the bottom: "are" strongly coupled

Reviewer #2 (Remarks to the Author):

I appreciate author's efforts to address the reviewers' comments. The revised manuscript is definitely better organized and the typos, mistakes, and inconsistencies seem to be fixed. The added clarifications and explanations make the paper an easier read and a bit more accessible for a wider audience. The Applications section has been extended and now contains a much better description of the experiments and a clear explanation of failures of traditional ET models to describe these experiments. However, I still think that the paper is too technical for Nature Communications and is more suitable for a specialized journal. As I mentioned earlier, the paper does not provide a direct comparison to the experimental kinetic data but rather suggests qualitative explanations of the observed kinetic behaviors using the model with some reasonably chosen parameters. That said and after reading the author's rebuttal letter I do recognize that the model can be regarded as a novel application of the well-known theoretical concepts to describe quite unique experiments on ultrafast ET in proteins. As I mentioned in my previous review, I do think that the paper can be of great interest to experimentalists and theoreticians in the field and it is up to the Editor to decide whether it is suitable for this particular journal.

Reviewer #3 (Remarks to the Author):

The authors have, in my opinion, addresses satisfactorily all the points raised by the referees in the previous round of review. I confirm my opinion concerning the quality and novelty of the work presented in the manuscript.

Several technical details have been corrected or clarified in the revised version and rebuttal letter. The author's response to the issues raised is indeed thorough and convincing. As recognized by all referees, the paper is scientifically sound and deserves publication.

In my opinion, the paper is suitable for publication in Nature Communication (in preference to a more specialized journal) for the following reasons:

(1) novelty: the method proposed in the paper is capable of addressing competing dynamical regimes not properly treatable with other approaches;

(2) physical insight: it is clear from the application presented in the paper that the method can be used not only as a phenomenological model of experimental data, but also as a means to identify competing factors affecting ET reactions in complex media; it would be very intriguing to see it extended to wide classes of systems;

(3) generality: in principle the model proposed is applicable to several classes of ET processes, not

limited to those occurring in biological systems; it could be, for instance, of potential major relevance in materials science and chemistry.

I therefore believe that the work presented in the manuscript will be of interest to wide research communities working in the field of ET processes and I recommend publication of the manuscript in its revised form.

Reviewer #4 (Remarks to the Author):

As in my earlier comments, I think the reported research is interesting and was well performed, but is quite technical and specialized. The responses to my earlier concerns do not justify the need for urgent publication in Nature Communication.

1. Author Respond: Frist, I fully appreciate the reviewer's time and efforts on our work. I have to emphasize here that we are not a theory group and we did not aim to create a new theory in this work. What we did is that we like to develop a practical ET model based on previous theories/models to explain our observed experimental results. Currently, we did not find a theory or model in literature that can perfectly explain our data. Thus, we combined two powerful, previous models and developed an extended model to explain our data. Then, from our systematic studies, we can extract insightful knowledge about the molecular mechanism of ultrafast ET reactions. In turn, we can improve our new model again. Here, we showed the nice extended model can truly explain our observed ultrafast ET reactions. Thus, we do think the paper is better suitable to Nature Communication due to the significance of the extended model that can be used to explain a variety of ET reactions in material, chemistry and biology."

As the authors stated this is "an extended model to explain our data" and I am not sure a single case study can be considered as "systematic studies". I did not see the potential for the use in "a variety of ET reactions in material, chemistry and biology".

2. Author Respond: The single-exponential decay in the frozen region is a result of the following assumptions:
a. Firstly, it is assumed that the electronic coupling between states J is a constant such that it does not vary with different configurations of the system.
b. Secondly, it is assumed that the configuration of the environment does not affect the sum of the driving force and the total reorganization energy, ΔGo + λ. This is a result of the assumption that the donor and acceptor interact with the environment through linear dipole-dipole interactions.
If either of these assumptions is broken, the ET dynamics in the frozen region will not be exponential. In the short-range ET reactions that we are concerned with in this work, these assumptions generally hold true. Moreover, by a few simple modifications, the nonergodic model can be applied to cases in which these assumptions are invalid.
This work helped us establish a different physical picture concerning the effects of local environment on the ET dynamics in the ultrafast regime. It has been understood that the free energy surface of a biological system (more generally, it can be any complex system) is rugged, with a hierarchy of energy barriers that result in multiple relaxation timescales (PNAS, 113, 30 (2016)). This is in contrast to the simple potential function of the reaction coordinate we usually assumed for an ET reaction, which puzzled us greatly. The nonergodic model helped us understand that the high energy barriers forbid samples that start at a certain part of the phase space from reaching other parts within a given time window (tw=1/kET). As a result, we can still use the reaction coordinate that has a simple potential function but with its curvature modified by the nonergodic effect.
We recognized the important contributions of related work (such as JCP, 103, 595, (1995) and Chem. Sci. 5, 3761, (2014)) to our understanding of nonequilibrium ET reactions. However, we think that the model in this work provides a novel and comprehensive description over ET dynamics on the ultrafast timescales, (the active and frozen regions). The description is also consistent with our experimental

results about which no satisfactory explanations have been made by far in literature.

I do not agree that there are only TWO assumptions (a. and b.) made to reach the conclusions (e.g. the choice of the distraction function). For the claimed generality, please demonstrate those woking conditions besides the single case study in the manuscript.

3. Author Respond: The decomposition of the reorganization energy and the reaction free energy is based on the relative timescales of various local motions as compared to the reaction timescale. Therefore, it is possible that the inner reorganization energy λi can include contributions from fast environmental motions that are not resolved by the solvation experiment. However, in flavodoxin, the functional site of flavodoxin is buried inside the binding pocket with partial exposure to the protein's surface (JACS, 132, 12741(2010)). The measured solvation dynamics around the functional site, including effects of surrounding motions up to 10 A˚, does not have sub-ps components, while typical ballistic motions are resolved in the ten of fs to hundreds of fs timescale (see for example Chem. Phys. Lett., 388, 120 (2004)). Hence, the fast, ballistic motions of water should not have important contributions to λi. We thus believe the decomposition of the reorganization energy is reasonable (also see reply to the reviewer 2 and see our hydration work).
On the other hand, we agree that the estimated value of for each mutant for FMN is questionable, since in this case γ and are both very small. Nevertheless, ≈ 0 does tell us the ET reaction is in the frozen region and the local environment barely moves during the reaction time, in comparison to the case of FMNH•. We think that even though the relevant parameters obtained by simulations, such as , , and Δ , may not be completely quantitatively accurate, this kinetic approach of modeling ultrafast ET dynamics has its value, and can be used to analyze experimental results and obtain very insightful information. Also, we cannot solve all questions in one paper. More work is underway to test our recent various ET reactions in different biological systems using the newly developed model.

My earlier comment is that the decomposition of the non-equilibriume effective reaction free energy barrier into contributions from the reaction free energy and the reorganization energy is not unique and cannot be general for ultrafast ET processes. The authors response essentially does not address this question. The analysis presented here is quite specialized for the system they investigated. Also, as the authors stated, "the estimated value for each mutant for FMN is questionable", should I trust the analysis based on those estimates?

4. Author Respond: Thanks for pointing it out. The sentence was not well phrased. We modified it in the revised manuscript. We did consider the potential roles of electronic and vibrational coherences, but we did not include these discussions into this paper. Firstly, "long-lived" electronic coherence often leads to oscillating population dynamics, but we did not observe any oscillating pattern in the data (Figure 5). Secondly, we argue that even if coherence is involved, the nonergodic effects on the ET dynamics still exist and the general conclusions about these effects should be qualitatively applicable to ET reactions in the frozen and active regions. Nonetheless, we are interested in examining the potential effects of coherence on ultrafast ET dynamics in the future work.

It's fine to consider the coherence effects on ultrafast ET dynamics in the future work. But, I do not agree with claim "the general conclusions about these effects should be qualitatively applicable to ET reactions in the frozen and active regions". I am not sure the formulation of coherent ET will be similar to those summarized in the manuscript.

Responses to review reports

REVIEWER 1

After reading the authors' rebuttal and the revised manuscript, I believe my concerns have been mostly addressed/explained. The problem studied here is obviously both important and immensely complex. The authors represent a world's leading group in ultrafast dynamics of protein electron transfer and the issues still not fully resolved in this study reflect the state of the art in this field as is seen by the front runners. I think that the analysis presented in this paper is a robust trade-off between the desire for a quantitative description of the kinetic data and a limited set of established parameters for this complex system. It provides other practitioners with an example of a successful and practical theoretical tool which can be implemented for other kinetic studies.

After reading the comments by other reviewers, I respectfully disagree with the notion that the theory application presented in this paper does not provide new theoretical insights. It is true that the basic theoretical concepts used in this paper have been laid out in the past. However, application of these general concepts to problems and systems as complex as protein electron transfer has been a very painful process. Even the Sumi-Marcus theory, which provided a very general framework for addressing competing time-scales back in 1986, has only recently started to find its applications in analyzing experiment. Clear-cut nonergodic effect in biology realistically have not been confirmed experimentally and, from this general standpoint of the state of the field, this paper presents a truly breakthrough development. There are potentially many more kinetic data falling into the realm of dynamic freezing and glassiness, which have not been appreciated as such, and still are waiting to be re-discovered because of the lack of direct and clear applications of abstract theoretical ideas to specific kinetic studies. I believe this paper paves the way to many such applications in the future. One has to realize that glassiness of proteins has been recognized starting from classical studies by Frauenfelder and Wolynes and has been in various ways entertained by a number of scholars. What does it mean practically in terms of specific rate measurements has been less clear. This paper offers exactly this perspective. I'm certain that many practitioners will find it more valuable than the abstract theoretical ideas entertained by theorists. The application of the theory to kinetic data also poses questions which future theories must answer and, in this way, also forces the theorists to refine their models (the most fruitful way the exchange of ideas should happen in science). The reviewers, in fact, raise some of these valuable suggestions pointing that this is not the end of the road and these results will help others to move forward.

Respond: We are immensely thankful for the reviewer's supportive comments. As it has been nicely summarized by the reviewer, it is until recently that the Sumi-Marcus model was successfully used to analyze experimental data of protein electron transfers (Science, 316, 747 (2007); Chem. Rev. 115, 11191 (2015)). We are still at an early stage to explore the effects of local motions with multiple timescales on ultrafast ET dynamics.

p. 25: The authors insist that the free energy of electron transfer should show some ruggedness when calculated "correctly". While this notion has been advocated in glass science, one has to realize that it has been applied to the configuration landscape. This is the potential energy of the system as a function of all 3N coordinates, and it must be rugged as evidenced by computer experiments. However, in the case of electron transfer, all 3N coordinates of the system are projected on a single collective electron-transfer reaction

coordinate. There is no evidence in the literature, including direct calculations of such free energy surfaces by molecular dynamics simulations, that this projection should preserve the ruggedness of the original $U(r_{3N})$ surface. The authors should either clearly indicate the evidence for such a claim or remove it altogether. The model used by the authors is already more complex than "simple diffusion" since the diffusion coefficient is time-dependent.

Theoretical work has shown that this model is equivalent to Langevin dynamics with stretched-exponential memory kernel. Do we have any present evidence that more complex models are required? I think this first paragraph on p.25 should be changed. The current write-up suggests that the model used in this paper is not adequate for this problem, while there is no evidence that this is true. In fact, the fitting of the experimental results presented in this paper suggests that the model is sufficient to describe ultra-fast electron transfer in proteins.

Respond: We appreciate the reviewer's kind suggestions. We are exploring the possibility that the existence of densely distributed local minima (i.e., "ruggedness") on the high-dimensional (r_{3N}) free energy surface of the system could introduce spatial and temporal disorders to the motion of the system along the projected reaction coordinate (see for example JPCB Lett. 110, 9363 (2006)). Related to this idea, entropic effects and the notion of transition state ensembles have been under active study in fields like protein folding and enzymatic catalysis (see for example Acc. Chem. Res. 48, 407 (2015)). In a sense, the projected free energy curve along a single reaction coordinate is rugged, although direct evidences are still lacking.

Specifically, the general formalism discussed by Wolynes (first introduced by Zwanzig) has the form of Fokker-Planck equations, subordinated to a temporal and spatial memory kernel (JCP, 78, 470 (1983); Phys. Rev. 124, 4 (1961)). Variations of this general kernel have been used to discuss anomalous dynamics in complex environments (see for example J. Phys.: Condens. Matter, 20, 373101 (2008)). However, in terms of the ultrafast ET dynamics, careful analysis needs to be carried out to justify the usage of more complex models.

Nevertheless, as the reviewer has commented, we have not obtained strong results showing that the diffusive description of local fluctuations is insufficient. The model used in this work makes a reasonable balance between practicality and sophistication in theory.

Minor corrections:

1. *p5, first par: remove break between sentences;*
2. *eq 9: the authors use 1 and 2 for the initial and final states, as well as R and P. It would be useful if they made connection between the two conventionsm such as 1=R, 2=P.*
3. *I suggest that the authors find ways to remind the reader the meaning of frozen/active/equilibrium through the manuscript. This is particularly true for those who are familiar with Marcus' normal/inverted classification. For instance, on p.23, "being in the active region" might follow with the reference like (Fig. 1B, II).*
4. *p. 23, 2nd line from the bottom: "are" strongly coupled.*

Respond: Thanks again for the careful review. These comments and suggestions helped greatly during our revision of the manuscript.

REVIEWER 2

I appreciate author's efforts to address the reviewers' comments. The revised manuscript is definitely better organized and the typos, mistakes, and inconsistencies seem to be fixed. The added clarifications and explanations make the paper an easier read and a bit more accessible for a wider audience. The Applications section has been extended and now contains a much better description of the experiments and a clear explanation of failures of traditional ET models to describe these experiments. However, I still think that the paper is too technical for Nature Communications and is more suitable for a specialized journal. As I mentioned earlier, the paper does not provide a direct comparison to the experimental kinetic data but rather suggests qualitative explanations of the observed kinetic behaviors using the model with some reasonably chosen parameters. That said and after reading the author's rebuttal letter I do recognize that the model can be regarded as a novel application of the well-known theoretical concepts to describe quite unique experiments on ultrafast ET in proteins. As I mentioned in my previous review, I do think that the paper can be of great interest to experimentalists and theoreticians in the field and it is up to the Editor to decide whether it is suitable for this particular journal.

Respond: We are very glad that the reviewer has recognized our work. The comments have been very helpful. We have made a lot of efforts on revising the manuscript. We worked hard to connect knowledge inside various fields and make the work accessible to general readers of Nature Communications.

REVIEWER 3

The authors have, in my opinion, addresses satisfactorily all the points raised by the referees in the previous round of review. I confirm my opinion concerning the quality and novelty of the work presented in the manuscript.

Several technical details have been corrected or clarified in the revised version and rebuttal letter. The author's response to the issues raised is indeed thorough and convincing. As recognized by all referees, the paper is scientifically sound and deserves publication.

In my opinion, the paper is suitable for publication in Nature Communication (in preference to a more specialized journal) for the following reasons:

1. *novelty: the method proposed in the paper is capable of addressing competing dynamical regimes not properly treatable with other approaches;*
2. *physical insight: it is clear from the application presented in the paper that the method can be used not only as a phenomenological model of experimental data, but also as a means to identify competing factors affecting ET reactions in complex media; it would be very intriguing to see it extended to wide classes of systems;*
3. *generality: in principle the model proposed is applicable to several classes of ET processes, not limited to those occurring in biological systems; it could be, for instance, of potential major relevance in materials science and chemistry.*

I therefore believe that the work presented in the manuscript will be of interest to wide research communities working in the field of ET processes and I recommend publication of the manuscript in its revised form.

Respond: We greatly appreciate the recognition of our work by the reviewer.

REVIEWER 4

As in my earlier comments, I think the reported research is interesting and was well performed, but is quite technical and specialized. The responses to my earlier concerns do not justify the need for urgent publication in Nature Communication.

As the authors stated this is "an extended model to explain our data" and I am not sure a single case study can be considered as "systematic studies". I did not see the potential for the use in "a variety of ET reactions in material, chemistry, and biology".

I do not agree that there are only TWO assumptions (a and b) made to reach the conclusions (e.g. the choice of the distraction function). For the claimed generality, please demonstrate those working conditions besides the single case study in the manuscript.

Respond: Our focus of this work is to understand the complex dynamics of protein electron transfer. Although the Sumi-Marcus model has been used to describe ET dynamics in the non-diffusion limit, i.e., the frozen region (see for example, Chem. Phys. 236, 355 (1998); JCP, 98, 1228 (1993)), these cases are basically the result of "static" heterogeneity. However, our work shows that the model is insufficient for describing ET dynamics in protein flavodoxin (frozen and active region). Similar dynamics has also been observed in other flavoproteins (PNAS, 98, 11867 (2001); JPCB, 106, 8917 (2002)). Ultrafast single-exponential ET dynamics that is possibly in the frozen region has been reported in LMCT (Ligand-Metal-Charge-Transfer) processes involving copper in laccases (Biophys. Chem. 200, 41 (2015)), Rusticyanin (JPCB, 116 4192 (2012)) and Plastocyanin (PCCP, 12, 6067 (2010)). We suggest that this type of dynamics is the result of the glassiness of protein solution (broken of ergodicity), which has also been pointed out by other reviewers. Therefore, it is very likely that more reactions that have been discovered in the past could belong to ET reactions in the frozen region while stay unnoticed.

Furthermore, given the universal existence of ergodicity breaking in complex environments, such as protein, DNA and other condensed-matter systems, we think our approach of modeling charge-transfer dynamics can be applied in a wider context. This is why we believe that this work is worth being published in Nature Communications such that it can provide other practitioners with the necessary tools to make more discoveries.

My earlier comment is that the decomposition of the non-equilibrium effective reaction free energy barrier into contributions from the reaction free energy and the reorganization energy is not unique and cannot be general for ultrafast ET processes. The authors response essentially does not address this question. The analysis presented here is quite specialized for the system they investigated. Also, as the authors stated, "the estimated value for each mutant for FMN is questionable", should I trust the analysis based on these estimates?

Respond: We think that we misunderstood the reviewer's question in the earlier comments. We were addressing instead the question concerning the decomposition of the total reorganization energy. Here, we would like to repeat the previous question that the reviewer asked in the following and respond to it again.

The overall nonergodic impact to kinetics is to reduce the effective reaction free energy barrier (J. Mol. Liq. 266, 361). The further assignment of the non-equilibrium reaction free energy (in principle not well defined) to contributions from the reorganization energy and

other components is somewhat empirical and depends on the detailed knowledge of dynamics of the system (e.g. solvent dynamics S(t) or S(ω) to define γ in the current formulation). These could introduce some arbitrariness in the application. For example, in Table I, λ_o^γ varies from 0.14eV to 0.33eV for FMN for different mutants. I am not sure this is valid. β from fitting the experiments is valid, and overall effective free energy barrier inferred from experiments is useful. The further decomposition may not be unique.

Respond: We don't agree with some of the reviewer's comments. It is now clear that using traditional ΔG^o and λ_o^{eq} to directly compute the ET rate of ultrafast ET reactions is not correct; the physics underlying ultrafast ET would be different. Here we introduce a dynamic factor γ to better quantify such effect, *i.e.*, the degree of nonergodicity, which is determined by the ET time constant and the solvent correlation function (S(t) or S(ω)). Furthermore, the assumption of linear coupling between the donor and acceptor leads to the relation between the reaction free energy and the reorganization energy (eq (20)), $\Delta G^\gamma = \Delta G^o + (1-\gamma)\lambda_o^{eq}$.

More specifically, the value of λ_o^{eq} of different mutant is determined by the intrinsic system. In Table 1, due to the very small value of γ and λ_o^γ for the oxidized state, a noticeable error could be introduced into λ_o^{eq} (not λ_o^γ in the reviewer's comment). In comparison, in the semiquinone case, the γ values are larger, compared to the values of the oxidized state, and the difference between values of λ_o^{eq} is not as large. Hence, the greater variation of the values of λ_o^{eq} for the oxidized state is partly a result of the very small values of γ and λ_o^γ with a rounded second decimal place. Such small values highly depend on the experimental accuracy of ET rate, S(t), and ΔG^o.

It is fine to consider the coherence effects on ultrafast ET dynamics in the future work. But, I do not agree with claim "the general conclusions about these effects should be qualitatively applicable to ET reactions in the frozen and active regions". I am not sure the formulation of coherent ET will be similar to those summarized in the manuscript.

Respond: Based on our limited knowledge, we think that the electronic coherence would not have significantly impacts on the dynamics of ET reactions coupled to a classical bath.

Firstly, as we have mentioned in the last letter of response, "long-lived" electronic coherence often leads to oscillating population dynamics, but we did not observe any oscillating patterns in the data (Figure 5). Hence, it is likely that the lifetime of coherence is much shorter than the reaction time.

Secondly, in another theoretical paper of ours that is currently under review, starting from the spin-boson model, we showed that under a quite general set of parameters that are reasonable in protein electron transfers, the electronic coherence is short-lived. We outline the relevant part in the following.

We start with the spin-boson model and assume that there exists a classical reaction coordinate (see for example JCP 83, 4491 (1985); JCP 87, 2090 (1987)). Then the spin-boson Hamiltonian can be canonical transformed to a new Hamiltonian with a reaction coordinate and a transformed harmonic bath. If we assume that the coupling between the ET system and the bath is small, the reduced density operator (i.e., the density operator of the ET system alone) follows the quantum master equation (similar approach was used in JCP 111, 2075 (1999)),

$$\frac{\partial}{\partial t}\rho(x,t) = \mathcal{L}\rho(x,t) - \frac{1}{2}k_D\{|2\rangle\langle 2|, \frac{\partial}{\partial x}\rho(x,t)\} - \frac{i}{\hbar}[H_S, \rho(x,t)],$$

Where H_S is the Hamiltonian of the ET system,

$$H_S = \Delta U_f\ |2\rangle\langle 2| + J(|1\rangle\langle 2| + |2\rangle\langle 1|),$$

and J is the electronic coupling between the two states. The curly bracket {A,B} stands for the anti-commutator of the two operators A and B. The energy of the state $|1\rangle$ is set to 0. The Liouville operator describing an overdamped Brownian particle under a harmonic potential is given by

$$\mathcal{L} = D\frac{\partial^2}{\partial x^2} + k_D\frac{\partial}{\partial x}x.$$

The reaction coordinate x has been rescaled such that the minimum of the state $|1\rangle$ is at $x = 0$, while that of the state $|2\rangle$ is at $x = 1$. k_D is the relaxation rate along the reaction coordinate. D is the diffusion coefficient along x, given by $D = \frac{k_B T k_D}{2\lambda_o}$.

The coherence, i.e., the off-diagonal element of $\rho(x,t)$, $\rho^{12}(x,t)$, follows the equation,

$$\frac{\partial}{\partial t}\rho^{12} = \left(D\frac{\partial^2}{\partial x^2} + k_D\frac{\partial}{\partial x}\left(x - \frac{1}{2}\right)\right)\rho^{12} + \frac{i}{\hbar}\left[\Delta U_f - 2\lambda_o x\right]\rho^{12} + \frac{i}{\hbar}J[\rho^1 - \rho^2].$$

We find that the following equation,

$$\frac{\partial}{\partial t}g = \left(D\frac{\partial^2}{\partial x^2} + k_D\frac{\partial}{\partial x}\left(x - \frac{1}{2}\right)\right)g + \frac{i}{\hbar}\left[\Delta U_f - 2\lambda_o x\right]g,$$

can be analytically solved by the method of characteristics (Risken, The Fokker-Planck Equation, Springer-Verlag). Given that the initial condition, $g(x, t = 0) = \delta(x - x_0)$, the solution of the equation reads,

$$g(x,t|x_0) = A(t)G(x,t|x_0),$$

where

$$A(t) = \exp\left[\frac{i}{\hbar}\left(\Delta U_f - \lambda_o\right)t\right]\exp\left[-k_0\left[t - \frac{1}{2k_D}(3 - e^{-k_D t})(1 - e^{-k_D t})\right]\right],$$

and

$$G(x,t|x_0) = \sqrt{\frac{k_D}{2\pi D(1 - e^{-2k_D t})}}\exp\left[-i\frac{2\lambda_o}{\hbar k_D}x_0(1 - e^{-k_D t})\right]$$

$$\times \exp\left\{-\frac{k_D}{2D}(1 - e^{-2k_D t})^{-1}\left[x - x_0 e^{-k_D t} + i\frac{k_B T}{\hbar k_D}(1 - e^{-k_D t})^2\right]^2\right\}$$

The constant k_0 in $A(t)$ is given by $k_0 = \frac{2\lambda_o k_B T}{\hbar^2 k_D}$.

If we take a look at the short-time behavior of $A(t)$, the real exponent in $A(t)$ scales as $O(t^3)$ in the short-time limit,

$$\exp\left[-k_0\left[t - \frac{1}{2k_D}(3 - e^{-k_D t})(1 - e^{-k_D t})\right]\right] \approx \exp\left[-k_0 \times \left(\frac{1}{2}k_D^2 t^3\right)\right] \equiv \exp[-(t/\tau_d)^3].$$

The time constant τ_d can be viewed as the lifetime of ρ^{12},

$$\tau_d = \left(\frac{\lambda_o k_B T k_D}{\hbar^2}\right)^{-\frac{1}{3}}.$$

Clearly, τ_d increases as k_D or λ_o decreases. We may choose values such as $\lambda_o = 0.01$eV and $k_D = \frac{1}{1\text{ps}} = 1\text{ps}^{-1}$, which gives $\tau_d \approx 120$fs.

Note that during the calculation, we did not include the classical component of intramolecular vibrational modes (i.e., the inner reorganization energy), which by itself is very significant and will further decrease the coherence lifetime. Thus, we conclude that the electronic coherence should not be an important contributing factor to the ET dynamics studied in this work.

Furthermore, given the short lifetime of coherence, we showed in our other paper that the quantum master equation of $\rho(x, t)$ can be reduced to the Sumi-Marcus model. If vibrational coherences are significant, the reduced density operator $\rho(x, t)$ acquires the dependence on vibrational quantum numbers. Since the effect of nonergodicity modifies the motion along the classical reaction coordinate that describes local fluctuations, our treatment in this work should be generally applicable to ET dynamics in complex environments. Nevertheless, we are eager to discuss with the reviewer about phenomena and results beyond our scope of knowledge.
